# Pericentromeric satellite lncRNAs are induced in cancer-associated fibroblasts and regulate their functions in lung tumorigenesis

**DOI:** 10.1038/s41419-023-05553-1

**Published:** 2023-01-12

**Authors:** Natella I. Enukashvily, Nikita V. Ponomartsev, Avanee Ketkar, Roman Suezov, Anna V. Chubar, Andrey D. Prjibelski, Daria D. Shafranskaya, Sabrina Elmshäuser, Corinna U. Keber, Vera N. Stefanova, Andrey L. Akopov, Ursula Klingmüller, Petra I. Pfefferle, Thorsten Stiewe, Matthias Lauth, Anna I. Brichkina

**Affiliations:** 1 Institute of Cytology, Russian Academy of Sciences, 194064 St.-Petersburg, Russia; 2 Institute of Molecular and Cell Biology, A*STAR, 138673 Singapore, Singapore; 3 Philipps University of Marburg, Department of Gastroenterology, Center for Tumor- and Immune Biology, 35043 Marburg, Germany; 4 Philipps University of Marburg, Institute of Molecular Oncology, 35043 Marburg, Germany; 5 Member of the German Center for Lung Research (DZL), Philipps University of Marburg, Marburg, Germany; 6 Center for Algorithmic Biotechnology, St.-Petersburg State University, 199034 St.-Petersburg, Russia; 7 Philipps University of Marburg, Institute of Pathology, 35043 Marburg, Germany; 8 Pavlov First State Medical University, 197022 St.-Petersburg, Russia; 9 German Cancer Research Center (DKFZ), 69120 Heidelberg, Germany; 10 Philipps University of Marburg, Comprehensive Biobank Marburg CBBMR, 35043 Marburg, Germany

**Keywords:** Cancer microenvironment, Non-small-cell lung cancer

## Abstract

The abnormal tumor microenvironment (TME) often dictates the therapeutic response of cancer to chemo- and immuno-therapy. Aberrant expression of pericentromeric satellite repeats has been reported for epithelial cancers, including lung cancer. However, the transcription of tandemly repetitive elements in stromal cells of the TME has been unappreciated, limiting the optimal use of satellite transcripts as biomarkers or anti-cancer targets. We found that transcription of pericentromeric satellite DNA (satDNA) in mouse and human lung adenocarcinoma was observed in cancer-associated fibroblasts (CAFs). In vivo, lung fibroblasts expressed pericentromeric satellite repeats HS2/HS3 specifically in tumors. In vitro, transcription of satDNA was induced in lung fibroblasts in response to TGFβ, IL1α, matrix stiffness, direct contact with tumor cells and treatment with chemotherapeutic drugs. Single-cell transcriptome analysis of human lung adenocarcinoma confirmed that CAFs were the cell type with the highest number of satellite transcripts. Human HS2/HS3 pericentromeric transcripts were detected in the nucleus, cytoplasm, extracellularly and co-localized with extracellular vesicles in situ in human biopsies and activated fibroblasts in vitro. The transcripts were transmitted into recipient cells and entered their nuclei. Knock-down of satellite transcripts in human lung fibroblasts attenuated cellular senescence and blocked the formation of an inflammatory CAFs phenotype which resulted in the inhibition of their pro-tumorigenic functions. In sum, our data suggest that satellite long non-coding (lnc) RNAs are induced in CAFs, regulate expression of inflammatory genes and can be secreted from the cells, which potentially might present a new element of cell-cell communication in the TME.

## Introduction

The central role of the tumor microenvironment (TME) in the initiation and progression of primary lung cancer has been recognized [1, 2]. It is now widely accepted that multiple soluble factors, tissue remodeling enzymes, extracellular matrix (ECM) and other effectors produced by the stromal cells create a favorable niche for the expansion of lung cancer cells [3–9]. Cancer-associated fibroblasts (CAFs) and tumor-associated macrophages (TAMs) are the central and most abundant cells in the TME involved in building the tumor stroma and critical for the execution of innate and T-cell mediated anti-cancer response. TAMs are highly heterogeneous and plastic cell components of the TME which can either promote tumor progression (M2-like) or boost antitumor immunity (M1-like) [10]. Like TAMs, CAFs appear to be a heterogeneous group of cells in lung cancer with different origins and functions [11, 12]. In solid tumors, ECM and soluble factors secreted by CAFs mediate cancer cell proliferation, metastasis, and resistance to therapy. CAFs contribute to the immune escape of tumors and directly abrogate the function of cytotoxic lymphocytes [13–16]. Secreted factors produced by CAFs in response to chemotherapy create a favorable niche for cancer cells to escape death and facilitate tumor relapse [17–19]. Hence, the identification of a broad set of protein and RNA-based factors expressed by TAMs and CAFs involved in cell-cell communication in the lung TME will be essential to aid the development of new treatments and diagnostic tools for cancer therapy.

Special attention is currently paid to cell-cell communication mediated by non-coding RNA. Long non-coding RNA (lncRNA) interacts with other cellular macromolecules, including DNA, protein, RNA and are involved in tumor/stroma crosstalk to stimulate a permissive TME [20, 21]. The role of lncRNA in oncogenic transformation and cancer progression is now well established, however mostly for lncRNA from the genome’s intergenic or intragenic regions [20]. Transcription of non-coding tandemly repeated (TR) DNA of centromeric and pericentromeric chromosome regions was demonstrated in different species, including human and mouse [22]. In human, the transcripts were characterized as lncRNAs transcribed from pericentromeric TR DNA belonging to human satellite families 1, 2 and 3 (HS1, HS2, HS3). HS2 and HS3 are based on ATTCC repeats and though closely related have distinct sequences and organization. In genus *Mus*, the most abundant TR DNA superfamily is a major satellite (MaSat) [23]. The MaSat sequence is based on a repeating unit of less than 20 bp in length, and four major oligonucleotides derived from an original sequence d(GA5TGA) were identified [24]. The human HS2/HS3 and mouse MaSat sequences, while not homologous, have similar functions with the same proteins binding to them [25, 26].

Aberrant overexpression of satellite repeats has been previously reported for epithelial cancers and cancer cell lines in vitro [27–30]. These observations were based on the analysis of resected mouse or human tumors, cultivated cancer cells in 3D conditions [31, 32], or acquired through a bioinformatics approach [31]. However, the examination of bulk tumor material or of only cancer cells in culture could mask the contribution of tumor stromal cells which can comprise a majority of tumor mass in lung adenocarcinoma and make a significant impact on data interpretation [33]. We aimed here to assess the expression and physiological role of pericentromeric satellite repetitive elements in lung cancer, with a particular focus on cells of the TME. Unexpectedly, we found that satDNA transcripts were strongly induced in CAFs, regulated their inflammatory pro-tumorigenic functions and could be located extracellularly. These findings open new avenues to consider satRNAs as a part of the secretory CAFs phenotype potentially involved in cell–cell crosstalk in the TME.

## Results

### Cancer-associated fibroblasts express satellite lncRNAs in lung cancer stroma

**Fig. 1:**
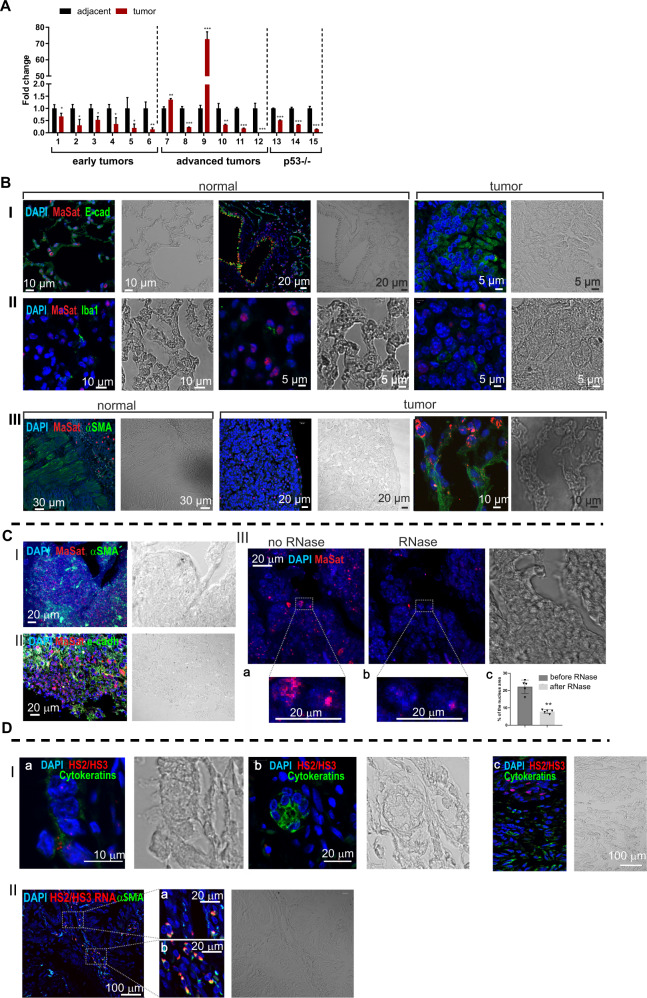
Transcription of satellite DNA in CAFs in mouse and human lung cancer. **A** RT-qPCR analysis of MaSat transcription in mouse lung tumors (red bars) and adjacent tissue (black bars). Cut tissues were obtained from mice bearing mutated *Kras*^*G12D*^ at the age of 10-14 weeks (early tumors), late stages with advanced lung cancer (33-47 weeks old), or from p53-deficient mice at the terminal stage of life. The numbers on the X-axis correspond to the individual mice (animal number). Each sample is a pool of ≥3 individual tumors or adjacent tissue from the same tumor lung. Data are mean ± SD. *p < 0.05; **p < 0.01; ***p < 0.001. **B** DNA-RNA FISH of MaSat transcripts on histological sections of lungs from Kras^G12D^ mice 10–14 weeks of age. MaSat transcripts (red), epithelial E-cadherin (green, panel I), macrophage marker Iba1 (green, panel II), fibroblast marker αSMA (green, panel III). Scale bars are shown in the images. **C**. DNA-RNA FISH of MaSat transcripts on histological sections of lungs from Kras^G12D^ 33–47-week-old mice. MaSat transcripts (red), fibroblast marker αSMA (green, panel I) epithelial marker E-cadherin (green, panel II). The images in panel III show the same field before (a) and after (b) RNase treatment (see material and methods; panel III). The hybridization signal could be removed almost completely by RNase treatment. Representative images (in dotted rectangles) of treated and untreated nuclei are shown at higher magnification. The nuclei in all images are counterstained by DAPI (blue). Scale bars are shown in the images. The results of RNase treatment quantification (see Supplemental material and methods, Microscopy section) of 5 randomized fields (cell number ∼150–200 per field) is shown in (panel III, c). *Y-axis*—percentage of the nucleus area occupied by FISH signals. Mean and standard deviations are plotted along with the individual values used for calculations. **p < 0.01. **D** Panel I. HS2/HS3 transcripts (red) in human adenocarcinoma sections stained with the CKMN AB specific to a wide spectrum of cytokeratines (green). The corresponding phase-contrast images are shown. (a) normal airway epithelium, (b) small adenocarcinoma nodule, (c) adenocarcinoma with papillary growth pattern. The nuclei are counterstained by DAPI (blue). Scale bars are shown in the images. Panel II. HS2/HS3 transcripts (red pseudocolor) in human adenocarcinoma sections stained with anti-αSMA antibodies (green pseudolor). The corresponding phase-contrast image is shown. At the right—fragments (a) and (b) represent the areas inside the dotted rectangles at higher magnification. The nuclei are counterstained by DAPI (blue). Scale bars are shown in the images. For each panel a representative image of no less than 10 scanned areas is shown.

To evaluate the expression of pericentromeric satellite repetitive elements in lung cancer, we used a mouse model with a somatic expression of mutated *Kras*^*G12D*^. We tested MaSat transcription in lungs with tumors and in healthy lungs (Fig. S1A) using the RT-qPCR method with random primers for cDNA synthesis. Surprisingly, the MаSat transcription level was lower in the tumor lesions than in the normal epithelium (Fig. S1B). Oligo-dT primers or random primers for reverse transcription (Fig. S1C) provided similar results as well as an RT-qPCR utilizing specific primers that were performed because the poly(A) protocol can fail to detect several classes of repeat RNA [30]. Reproducibly, we observed a decline in the transcription of MaSat in tumor tissue in 13 out 15 tested mice, independent of the stage or the status of p53 (Fig. 1A). Notably, in two tumor samples from mice at the terminal stage of the disease, a burst of MaSat transcription occurred (Fig. 1A, samples 7 and 9). These data suggest that the level of MaSat transcripts in most cases declines in lung tumors and depends on how advanced the cancer is and probably on the cell composition of the TME.

Next, we evaluated the distribution of MaSat transcripts on histological sections of mouse lungs with early tumors by the DNA-RNA FISH method. The oligonucleotide probe designed for DNA-FISH experiments marked all of the chromosomes in the metaphase plate (Fig. S2, panel I, a), and hybridized specifically to interphase condensed heterochromatin regions brightly stained by DAPI—chromocenters (Fig. S2, panel I, b)—a typical pattern of MaSat DNA distribution [34–36]. The RNA hybridization signals were detected mostly in peritumoral tissue outside the lesions. The MaSat transcripts were located within the nucleus, but outside chromocenters though close to them (Fig. S2, panel I, c). No signal was detected in RNA-FISH of RNase-treated sections (Fig. S2, panel I, d).

The MaSat transcription occurred mostly in normal epithelial cells stained with an E-cadherin antibody (Fig. 1B, panel I left and middle images). In contrast, little or no signals were detected in α-smooth muscle actin (αSMA)-positive fibroblasts of healthy tissues that underlie the airways epithelium (Fig. 1B, panel III, left image). In the tumor lesions, neither Iba1-positive macrophages infiltrating the tumor nor the cancer epithelial cells themselves contained MaSat hybridization signals (Fig. 1B, panels I and II, right images). The αSMA-positive spindle-shaped fibroblasts, typical for CAFs, at the periphery of the tumor were strongly stained with MaSat probed sequence (Fig. 1B, panel III). Thus, cancer cells from tumor nodules do not transcribe pericentromeric TR MaSat. Unlike normal fibroblasts, most CAFs surrounding the lesions transcribe the probed sequence.

High transcription level of satDNA has been previously reported in lung cancer [29]. We found induction of MaSat transcription only in 2 out of 6 samples from old mice with advanced adenocarcinoma (Fig. 1A). Late-stage tumors might display an onset of adenocarcinoma enriched with fibrous tissue that can significantly impact on gene profile expression [37–39]. Indeed, immune RNA-FISH staining of advanced lung cancer showed that lung tumors were highly abundant with MaSat positive cells, which were not only αSMA fibroblasts (Fig. 1C, panel I), but also E-cadherin epithelial cells (Fig. 1C, panel II). RNase-treated tissues were negative for satellite RNA signal (Fig. 1C, panel III), confirming specificity of the staining. These data suggest that satDNA is transcribed in cancer-associated fibroblasts and also in cancer cells at the later stages of lung tumorigenesis.

**Fig. 2:**
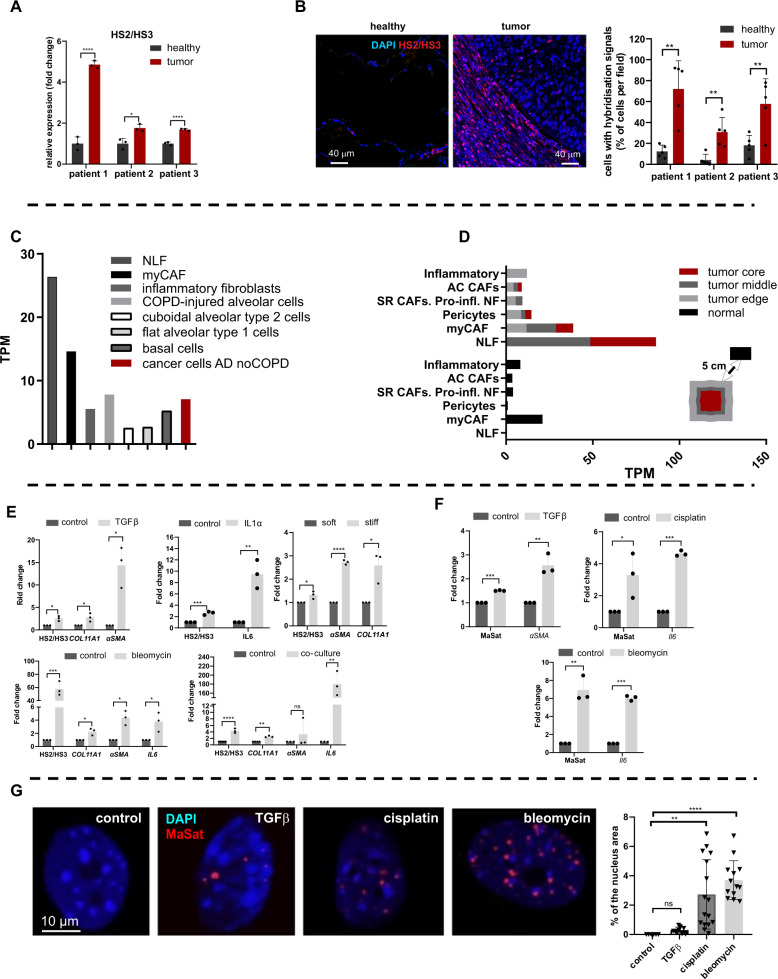
Transcription of satellite DNA is induced in lung cancer-associated fibroblasts in vitro. **A** RT-qPCR analysis of HS2/HS3 transcripts in bulk tumor tissue and corresponsing healthy adjacent tissue of three NSCLC patients. **B** Sections of healthy adjacent (left image) and tumor (right image) tissues probed with HS2/HS3 (red). The nuclei in all images are counterstained by DAPI (blue). Scale bars (40 µm) are shown in the images. Total magnification—×400. The results of DNA-RNA FISH quantification for three patients are shown in the graph. Five fields of view were taken for the quantification of each slide. **C** HS2/HS3 transcript quantity in NLF—normal lung fibroblasts, myCAF, inflammatory fibroblasts, epithelial and cancer cells (adenocarcinoma without COPD) was calculated for transcriptomes that we reassembled from single-cell RNAseq raw data published by Lambrechts et al. [11]. Quantification was performed for each cell cluster as a whole regardless of its origin from normal or tumor tissue. Data of quantification is shown as TPM value plotted on the *Y*-axis. **D** HS2/HS3 content in fibroblast subpopulation transcriptomes was calculated for cells obtained from the tumor core, middle and edge area, and non-malignant (‘normal’) adjacent tissue from the same resection specimen at maximal distance (>5 cm) from the tumor. Stress-response CAF—SR CAFs; proinflammatory normal fibroblasts—Pro-iNF; activated CAF—AC CAFs. Markers are shown in Table S1**. E** qRT-qPCR analysis of HS2/HS3 transcripts and CAF markers as positive controls in serum-starved and activated human lung fibroblasts (HFL1) untreated (control) or treated for 2 days with 10 ng/ml TGFβ1, 10 ng/ml IL1α, 30 μg/ml bleomycin, co-cultured with human lung cancer cells PC-9 or grown on soft and stiff matrixes. Data are mean from three independent experiments ± SD (*n* = 3). *p < 0.05; **p < 0.01; ***p < 0.001, **F** RNA level of MaSat or marker genes *αSMA* or *IL6* in primary serum-starved mouse lung fibroblasts treated with 20 ng/ml TGFβ1 (left graph) or with 2 μg/ml cisplatin or 30 μg/ml bleomycin; *n* = 3. *Y*-axis—fold change. Data are mean from three independent experiments ± SD. ***p < 0.001. **G** MaSat transcripts were visualized by DNA-RNA FISH (red) in primary mouse lung fibroblasts as in (**F**). Representative nuclei are shown. The results of quantification of ≥ 12 fields (Materials and methods, Microscopy section) are shown in the graph. The nuclei are counterstained by DAPI (blue).

Next, we validated the expression of repetitive elements in patients diagnosed with no Chronic Obstructive Pulmonary Disease (COPD) lung adenocarcinoma. HS2/HS3 transcripts were visualized by DNA-RNA FISH with a probe that shared homology with satellites HS2 and HS3 (HS2/HS3) and could hybridize with pericentromeric regions of most chromosomes (Fig. S2, panel II, a). However, unlike mouse MaSat, the transcripts were located either inside the nucleus or extranuclear (Fig. S2, panel II, b, c) and were not observed in RNase-treated samples (Fig. S2, panel II, d). The hybridization signals were not detected in pan-cytokeratin positive adenocarcinoma cells (Fig. 1D panel I). Like in mouse lung cancer, many cells with HS2/HS3 transcripts were co-localized with fibroblast markers αSMA (Fig. 1D, panel II) or vimentin (Fig. S3A). Some of the transcripts were revealed in CD90 + /CD44 + positive mesenchymal stromal cells within the tumor (Fig. S3B), which are known to change their phenotype into CAF-myofibroblasts [40]. Human CD68 + macrophages did not hybridize with the HS2/HS3 probe (Fig. S3C). Importantly, in contrast to mouse MaSat DNA, transcription of satellite HS2/HS3 DNA, tested by RT-qPCR (Fig. 2A) and by DNA-RNA-FISH (Fig. 2B) in material from three NSCLC patients, is highly enhanced in lung tumor tissue versus adjacent corresponding healthy lung tissue (Fig. 2A, B). In both mouse and human lung adenocarcinomas, the pericentromeric satDNA transcription occurs predominantly in fibroblasts. However, human HS2/HS3 transcripts, but not mouse MaSat, were also located extranuclearly (Fig. S2, panel II, c; Fig. S3D, E). These data opened the hypothesis that pericentromeric satellite transcripts are an oncogenic promoter in human NSCLC and the transcripts could be secreted outside of the human cells.

### Computational analysis of HS2/HS3 expression in single cells from Non-Small Cell Lung Cancer (NSCLC)

Lung fibroblasts are very heterogeneous both in the normal lung as well as in lungs of patients with pulmonary diseases [11, 12, 41, 42]. We developed a new algorithm (see the “Materials and Methods” section) and analyzed single-cell transcriptomes of fibroblasts from resected NSCLC tissue published by Lambrechts et al. 2018 [11] to understand which population demonstrates the highest rate of input in HS2/HS3 transcription (Table S1; Fig. 2C). Four HS2/HS3 transcripts were found using the algorithm (Table S2). The maximal transcripts per million value (TPM) in patients diagnosed with lung adenocarcinoma were obtained for transcriptomes of normal lung fibroblasts (cluster 6 in Table S1) and CAFs with myofibroblast phenotype (myCAFs, cluster 1 in Table S1; Fig. 2C, Fig. S4A). However, normal lung fibroblasts transcribed satDNA only when they were a part of tumor stroma, but not in healthy tissue (Fig. 2D). In contrast, myCAFs from normal tissue contained transcripts, though in lesser quantity than myCAFs from tumor stroma (Fig. 2D). TPM value was higher for transcriptomes of fibroblasts from the tumor middle and edge parts than from the tumor core (Fig. 2D). In general, all fibroblasts obtained from the tumor-adjacent and, to a lesser extent, tumor core regions contained more HS2/HS3 transcripts than fibroblasts from normal tissues (Fig. 2D; Fig. S4A). In normal lung tissue, myCAFs and inflammatory CAFs (iCAFs) transcribed satellites at 2-5 times higher level than other fibroblast subpopulations. Thus, CAFs are the major source of satellite RNAs in lung cancer, which correlates with our immunohistochemistry data on lung tissues.

TPM values were variable between different types of cancer cells. The lowest level of HS2/HS3 transcripts was observed in cancer cells of patients with adenocarcinoma without COPD (Fig. S4A). The computational analysis provided evidence that myCAF and ‘normal’ fibroblasts were major cell populations in lung adenocarcinoma expressing HS2/HS3, higher than cancer cells from adenocarcinoma patients (no COPD). Noteworthy, COPD, associated either with adenocarcinoma or squamous carcinoma, correlated with high expression of HS2/HS3 in epithelial cells and cancer cells of epithelial origin (Fig. S4A). In contrast to adenocarcinoma, squamous carcinoma cancer epithelial cells were strongly positive for HS2/HS3 transcripts and co-localized with cytokeratins (Fig. S4B), which is also coherent with the bioinformatics data (Fig. S4A). Thus the level of transcription in tumor cells depends on the type of lung cancer and its association with COPD.

### Transcription of satellite DNA is induced in lung cancer-associated fibroblasts

The conversion of stromal fibroblasts into CAFs is a critical step in re-programming the normal microenvironment to create a cancer niche. Induction of CAF phenotype in lung fibroblasts could be recapitulated in vitro by treatment with various factors. For an in vitro model of activated fibroblasts, we used human lung fibroblasts HFL1. CAF subpopulations are described to include myofibroblastic and inflammatory phenotypes [12, 43, 44]. To re-program normal fibroblasts into CAFs, HFL1 cells were either treated with TGFβ1 (myCAFs), or with IL1α to activate them into iCAFs, or were co-cultured with the lung adenocarcinoma cells. In all conditions, HS2/HS3 expression was significantly induced in human CAFs (Fig. 2E). The expression was accompanied by the upregulation of ECM gene *COL11A1* for myCAFs or *IL6* for iCAF. Many chemotherapy drugs also induce the CAF phenotype associated with senescence and secretory features supporting tumorigenesis [19, 45]. Exposure of human fibroblasts to bleomycin, which is used in a model of senescence of fibroblasts and also in COPD, led to increased pericentromeric satellites transcription (Fig. 2E). We also utilized an established in vitro model of altered substrate mechanics by culturing HFL1 cells on collagen I-coated hydrogels of varying stiffness, either 0.2 kPa (mimicking soft or healthy conditions) or 50 kPa (mimicking stiff or fibrotic conditions) [46, 47]. We found that increased substrate stiffness also correlated with the induction of HS2/HS3 transcription (Fig. 2E, right graph). However, the level of HS2/HS3 transcripts was similar between primary fibroblasts established from a resected tumor of adenocarcinoma patient and its matching fibroblasts from a healthy lung lobe (two patients: Fig. S4C). Perhaps a loss of environmental niche and exposure to stress in culture [27] lead to the equilibration of satellite transcription in primary cultures of fibroblasts. Similar induction of mouse satellite transcripts was found in primary mouse lung fibroblasts in response to TGFβ and cytostatics cisplatin and bleomycin (Fig. 2F). In treated cells, the MaSat probe revealed numerous intranuclear signals of different sizes (0.2–2 µm) detected by DNA-RNA FISH. The number of signals was highest in bleomycin-treated cells (Fig. 2G), which correlated with the qPCR data (Fig. 2F).

The trans-differentiation of epithelial cells can also give rise to a CAF-like hybrid cell population which undergo epithelial-to-mesenchymal transition (EMT). The high rate of satellite transcription in some advanced lung cancers (Fig. 1B) might also be a consequence of EMT. However, despite obvious induction of EMT markers *ZEB1* and *SLUG* in human lung adenocarcinoma cell lines stimulated with TGFβ, transcription of HS2/HS3 was reduced. (Fig. S5). Taken together, these data demonstrate that HS2/HS3 are expressed selectively in stromal fibroblasts upon conversion to CAFs in response to different stimuli.

### Activated macrophages do not contribute to increased satellite transcription

Besides CAFs, tumor-associated macrophages (TAMs) comprise a major part of the TME and also dictate the outcome of an antitumor immune response. We analyzed the available transcriptome data of myeloid cells, isolated either from a normal lung biopsy or tumor tissue of adenocarcinoma patients published in Lambrechts et al. [11]. 8 clusters out of 12 demonstrated countable transcription of HS2/HS3 (Tables S3, S4). Five clusters of the myeloid cell populations (M1- and alveolar macrophages, dendritic cells, Langerhans cells) showed lower TPM values in tumor tissue than in normal lung (Fig. S6A). TPM values were slightly increased in the other two clusters of macrophages with no annotated markers, suggesting that some populations of TAMs might display upregulation of HS2/HS3 transcription. In vitro analysis of differentiation and activation of macrophages obtained from human peripheral blood mononuclear cells showed that the level of HS2/HS3 transcripts were declined by day 8 of differentiation from monocytes to macrophages (Fig. S6B) and also in response to conditioned media (CM) from human lung cancer cells or to a combination of IFNγ and lipopolysaccharide LPS (M1-like subtype). In response to IL4 (to mimic pro-tumorigenic M2-like subtype), the level of transcripts was unchanged (Fig. S6B). Similar results we obtained for mouse bone marrow-derived macrophages (Fig. S6C). In order to explore a discrepancy in in vitro data for macrophages activation and two clusters with high TPM number identified by a bioinformatics approach, we co-cultured human macrophages with primary lung cancer cells, and only in these conditions transcription of pericentromeric RNA was induced significantly in all three donors tested (Fig. S6B, right graph). We hypothesized that perhaps direct contact of macrophages with tumor cells leads to more pro-tumorigenic M2-like polarization of macrophages than treatment with conditioned media. We tested a set of M2 genes, inflammatory genes, IFN-response genes and genes involved in T-cell response. All genes except IRF1-pathway were strongly upregulated in co-cultivated macrophages compared to those treated with condition media (Fig. S7). This might explain a correlation with higher expression of HS2/HS3 in this condition. We do not exclude that the population of macrophages in the tumor stroma might be very heterogenous, and HS2/HS3 transcripts mark only certain types of TAMs. However, it is unlikely that activated macrophages in the tumor stroma contribute significantly to the overall transcription level of pericentromeric DNA.

### HS2/HS3 transcripts are co-localized with CD63-positive extracellular vesicles in CAFs and can be transferred to recipient cells

**Fig. 3:**
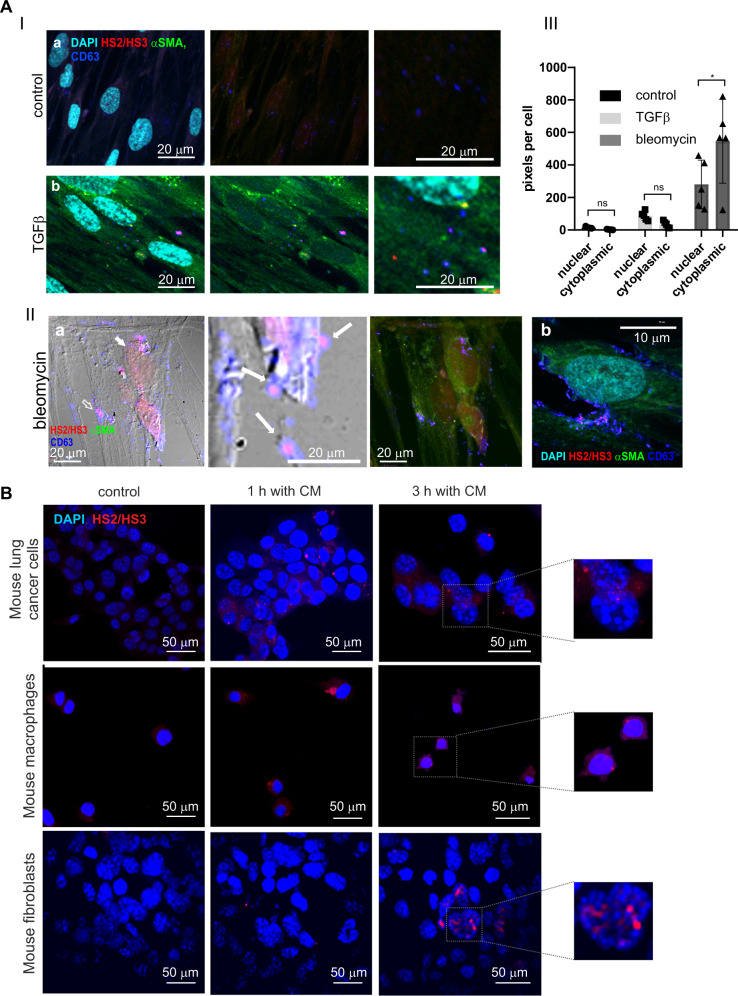
Extracellular localization of HS2/HS3 satellite transcripts in activated human lung fibroblasts and human HS2/HS3 transfer to mouse cells. **A Panel I**. HS2/HS3 transcription in serum-starved HFL1 fibroblasts left untreated (a) or after treatment for 4 d with 20 ng/ml TGFβ1 (b). Fixed fibroblasts were co-stained with HS2/HS3 probe (red), anti-αSMA antibodies (green) and CD63 (extracellular vesicles marker, blue). The nuclei are counterstained with DAPI (turquoise). Right image - intracellular CD63+ vesicles at higher magnification. Scale bars (20 μm) are shown in the images. Total magnification – x400. The results of immuno-FISH quantification of ≥12 fields are shown in the graph. **Panel II**. HS2/HS3 transcription (red) in serum-starved HFL1 fibroblasts after treatment with bleomycin. Cells were co-stained with anti-αSMA (green) and anti-CD63 (blue) antibodies. (*a*) cells with the intranuclear-cytoplasmic pattern of HS2/HS3 transcripts distribution; (*b*) A cell with cytoplasmic pattern of HS2/HS3 transcripts distribution. The nuclei are counterstained by DAPI (turquoise). Scale bars—**(*Ia*)** 20 µm, **(*IIb*)**—10 µm. Total magnification – x400. **Panel III**. The FISH images (5 randomized fields with cell number ≥150 in each of them) were quantified (see Supplemental material and methods, Microscopy section) to access the ratio between nucleoplasmic and cytoplasmic signals. The number of pixels occupied by FISH signals in cytoplasm and nuclei was calculated and plotted on the *Y-axis* Mean and standard deviations are plotted along with the individual values used for calculations. ns—non-significant (*p* > 0.05), *p < 0.05. **B** Mouse lung cancer cells, mouse BMD macrophages and MEF cells were serum-starved for 2 d and left untreated (control) or treated for 1 h or 3 h with conditioned media collected from bleomycin-treated serum-starved human HFL1 cells. Human HS2/HS3 transcripts were visualized by DNA-RNA FISH with DYZ probe (red). Representative images (in dotted rectangles) of cells after 3 h treatment are shown at higher magnification for better presentation of human HS2/HS3 intranuclear signals in mouse cells. For each panel a representative image of no less than 10 scanned areas is shown. The nuclei are counterstained by DAPI (blue). Scale bars 50 µm.

In most publications, including our own, only intranuclear localization of HS2/HS3 long non-coding RNAs was reported [27, 48, 49]. Therefore, we validated the subcellular localization of pericentromeric satellite transcripts in normal human lung fibroblasts HFL1 treated with TGFβ (Fig. 3A, panel I) or with bleomycin (Fig. 3A, panel II). Transcription of HS2/HS3 occurred in αSMA-positive cells both after TGFβ or bleomycin treatment (Fig. 3A). Treatment of HFL1 cells with bleomycin was the most potent inducer of HS2/HS3 transcription in cultured human fibroblasts (Fig. 3A, panel II). In some cells, the transcripts were detected in the cytoplasm or in close proximity to the nucleus (Fig. 3A, panel I,b). In other cells, all hybridization signals were localized in CD63-positive vesicles underlying the cell membrane (Fig. 3A, panel II, b). Some of the CD63-positive vesicles containing HS2/HS3 signal were localized outside the cells in the extracellular space (Fig. 3A, panel II, b). These data suggest that at least some of the HS2/HS3 transcripts could be localized outside the nucleus and exported from fibroblasts as cargo of extracellular vesicles (EVs).

To evaluate if secreted HS2/HS3 transcripts could be transferred from activated fibroblasts to other cells in the tumor, we incubated mouse cells of different origins with conditioned media collected from bleomycin-treated human fibroblasts. Human satellite HS2/HS3 RNAs were visualized by the FISH method. HS2/HS3 transcripts appeared in the cytoplasm of mouse macrophages, fibroblasts and tumor cells 1 h after the application of conditioned media from activated HFL1 and was present in cells for at least 3 h (Fig. 3B). These data demonstrate possible cell-to-cell transfer of secreted satellite transcripts. In cancer cells and in fibroblasts, some of the transcripts were revealed inside the nuclei (Fig. 3B, right images) suggesting the existence of cytoplasm-nuclei transport mechanisms for HS2/HS3 RNAs.

### Depletion of HS2/HS3 transcripts leads to rejuvenation of human fibroblasts

**Fig. 4:**
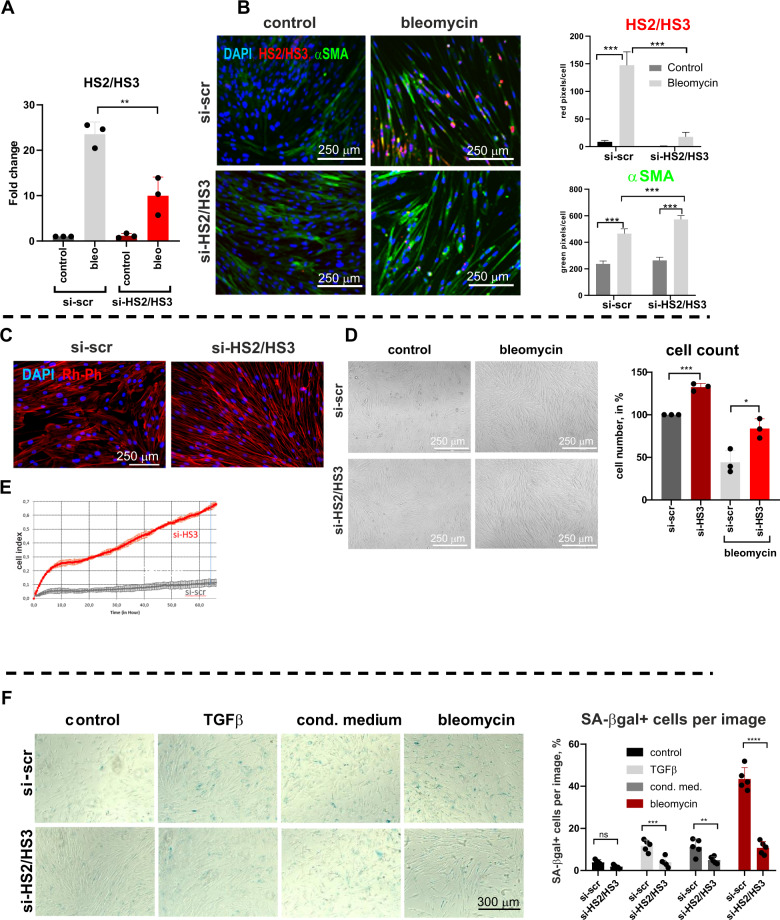
Knock-down of HS2/HS3 attenuates senescence of human lung fibroblasts. qRT-PCR analysis (**A**) or immune DNA-RNA FISH (**B**) of HS2/HS3 (red) and αSMA (green) in HFL1 transfected with scrambled siRNA or siRNA targeting human HS2/HS3 were serum starved and left untreated or treated for 4 d with 30 μg/ml bleomycin. Mean/SD from three independent experiments is shown. **C** HFL1 cells were transfected with siRNA, serum-starved for 6 days and stained with rhodamine-phalloidin (Rh-Ph). One out of five representative images is shown. **D** Brightfield image of HFL1 cells 6 d after transfection with siRNAs, serum starved untreated or treated with bleomycin. Cell number of cells grown for 4 d at 0.5% FCS media is presented at the graph (right); *n* = 3, 3 independent experiments. **E** xCELLigence real-time cell analysis (RTCA) dual purpose (DP) system (Agilent, USA) -recorded growth of primary human adult lung fibroblasts transfected with si-scr or si-HS2/HS3 and treated with bleomycin. **F** SA-βgal staining of starved HFL1 cells treated for 4 d with TGFβ, conditioned media from A549 tumor cells or 30 μg/ml bleomycin. One out of five representative images for each treatment is shown. Percentage of SA-βgal-positive cells per image has been calculated from 5 independent images and shown in the graph. Scale bars are shown in the images.

Human satellites are heterogenic, chromosome-specific, and their full sequences are not assembled yet [50]. Therefore there is no universal sequence to select for silencing. However, we managed to design siRNA oligonucleotides for partial knock-down of human HS2/HS3 which was confirmed by qPCR (Fig. 4A) and RNA FISH (Fig. 4B) in bleomycin-treated fibroblasts. We noticed that HFL1 cells deficient for HS2/HS3 adopted a more elongated morphology (Fig. 4B–D) which was associated with intensive αSMA staining (Fig. 4B) and with the formation of numerous elongated F-Actin fibers (Fig. 4C). Unexpectedly, depletion of HS2/HS3 also increased the proliferative potential of human fibroblasts grown under serum starvation conditions, reflected in higher density and increased number of cells (Fig. 4D). Similarly, primary human adult lung fibroblasts showed an advantage in proliferation when HS2/HS3 transcripts were depleted (Fig. 4E). It is known that primary human fibroblasts undergo senescence during passaging and a CAF secretory phenotype is also associated with senescence [45]. Transcription of pericentromeric satDNA was previously demonstrated to occur during cell senescence in various cell models [28]. To this end, we showed that human fibroblasts undergo cellular senescence in response to stimulating factors or chemotherapeutic drugs, detectable by the appearance of SA-βgal-positive cells (Fig. 4F) and visible by slightly more flat morphology and irregular cell shape (Fig. 4C). Depletion of satellite transcripts reduced the number of senescent human fibroblasts in growing conditions and after treatments (Fig. 4F). Collectively, these data reveal that satellite transcripts are involved in building the senescent phenotype of CAFs. Knock-down of satellite repetitive elements attenuates cellular senescence of human fibroblasts associated with the appearance of myofibroblastic features.

### Pericentromeric satellite lncRNAs regulate inflammatory functions of CAFs

**Fig. 5:**
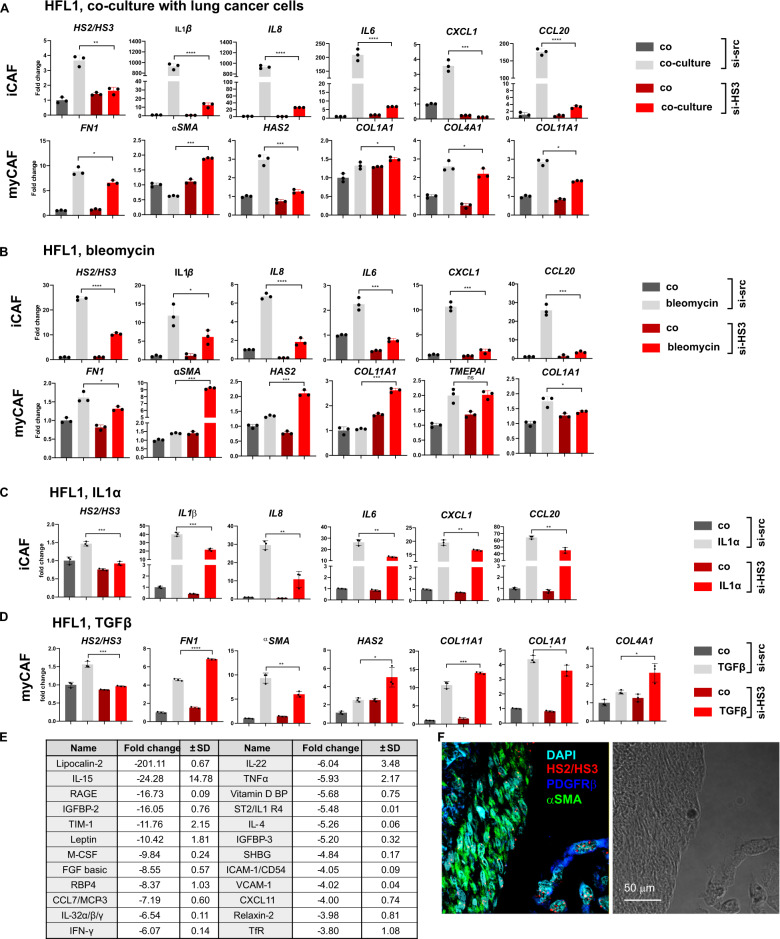
HS2/HS3 satellite transcripts are involved in building inflammatory phenotype of CAFs. qRT-PCR analysis of iCAF and myCAF genes in HFL1 cells transfected with scrambled siRNA or siHS2/HS3 serum-starved and co-cultured with PC-9 tumor cells (**A**) or treated with 30 μg/ml bleomycin for 4 d (**B**) or with 10 ng/ml IL1α (**C**) or 10 ng/ml TGFβ1 (**D**). *Y*-axis—fold change of relative expression (*n* = 3; mean ± SD). Data of one out of three independent experiments are shown. *p < 0.05; **p < 0.01; ***p < 0.001. **E** Summary of cytokine/chemokine level quantification in conditioned media collected from serum-starved si-scrambled or si-HS2/HS3 transfected HFL1 and treated with bleomycin for 4 d. Fold change and SD has been obtained as ratio of relative levels in si-scrabled sample versus si-HS2/HS3 transfected sample. None of cytokines was found to be upregulated in si-HS2/HS3 versus si-scrambled fibroblasts. Original membrane image is presented at Fig. S8A. **F** HS2/HS3 transcripts (red) in human adenocarcinoma sections stained with αSMA (myCAF, green) or PDGFRβ (iCAF, green).

CAFs are known to include distinct subpopulations with myofibroblastic and inflammatory phenotypes in adenocarcinomas [12, 43, 44]. The inflammatory (iCAF) subset is characterized by a secretory phenotype potentially regulating immune suppression; a myofibroblastic (myCAF) subset is characterized by αSMA expression, TGFβ-signaling and extracellular matrix production. We employed direct coculture of human HFL1 fibroblasts with lung cancer cells to understand how satellite transcripts affect polarization of lung fibroblasts. Before seeding cancer cells, HFL1 was transfected either with scrambled siRNA or with siRNA targeting HS2/HS3. Several previously described markers of iCAFs and myCAFs were tested by qPCR [12, 43, 44]. Remarkably, HFL1 with depleted satellite RNAs were not able to induce transcription of many inflammatory genes (e.g. *IL6*, *IL8*, *IL1β*, *CXCL1*) in coculture with lung cancer cells (Fig. 5A), whereas expression of myCAF genes (*COL1A1, COL4A1, αSMA, HAS2*, *FN1*, *COL11A1*) did not differ much between control HFL1 and si-HS2/HS3 transfected. Previous studies showed that cellular stress, including cytostatics, can induce an inflammatory fibroblast phenotype [45, 51]. We found also that in HS2/HS3-deficient fibroblasts, inflammatory genes, but not myCAF genes, were weakly activated in response to bleomycin (Fig. 5B), similar to coculture conditions. These data suggest that satellite transcripts mostly involved in the formation of inflammatory phenotype of activated fibroblasts, but do not affect the transcription of ECM genes marking myCAFs. To further validate this finding, the same set of inflammatory genes has been tested in IL1α-activated fibroblasts. Indeed, again knock-down of HS2/HS3 transcripts reduced an amplitude of inflammatory gene expression (Fig. 5C); whereas the expression level of myCAF marker genes induced by TGFβ in HFL1 cells did not depend on the depletion of satRNAs (Fig. 5D). In addition to RNA expression analysis, 28 cytokines/chemokines out of 106 tested by proteome array were significantly less present (difference is between 3.8 and 222.9 times) in conditioned media from bleomycin-treated HS2/HS3-deficient fibroblasts than from control si-scr cells (Fig. 5E, Fig. S8). Of note, none of the tested cytokines showed higher amounts in HFL1 with knock-down of HS2/HS3 in comparison to control si-scr cells (Fig. S8) suggesting that satellite repeats indeed regulated the transcription and secretion of many inflammatory cytokines/chemokines. Importantly, in situ myCAFs (αSMA+ cells) and inflammatory iCAFs (PDGFRβ+ cells) are two distinct populations of CAFs that transcribe satDNA in human lung adenocarcinoma tissue (Fig. 5F). All together, these data demonstrated that satellite transcripts mainly drive the inflammatory pro-tumorigenic phenotype of cancer-associated fibroblasts, but were less involved in myCAF functions.

### Pericentromeric satellite lncRNAs regulate pro-tumorigenic functions of CAFs

**Fig. 6:**
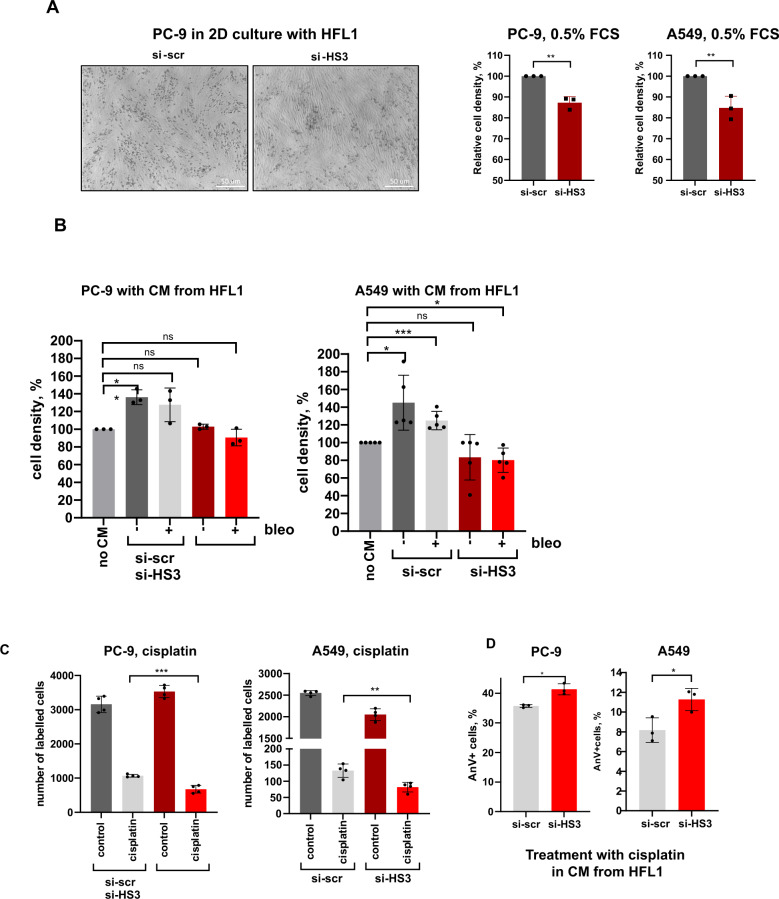
HS2/HS3 satellite transcripts regulate pro-tumorigenic functions of CAFs. **A** A549 and PC-9 cells were grown for 4 d in 0.5% FSC media in 2D contact coculture with HFL1 cells transfected either with si-scrambled (si-scr) or si-HS2/HS3 (si-HS3) RNAs. A representative photo for coculture of PC-9 cells with distinct round morphology is included. Number of tumor cells (measured as relative cell density) from 3 independent experiments is shown at the graph (absolute number of cells from one representative experiment is in Fig. S9A). **B** Serum-starved A549 or PC-9 tumor cells were grown in the presence of conditioned media collected from activated HFL1 cells or pre-treated with bleomycin. Relative cell number (%) from three (PC-9) and five (A549) independent experiments is presented. The photo of cells is in Fig. S9B, the absolute number of cells from one representative experiment is in Fig. S9C. **C**. A549 and PC-9 cells were grown for 4 d in 2D-coculture with HFL1 cells transfected either with si-scr or si-HS2/HS3 in the presence of 1 μg/ml cisplatin in media with 5% FCS. The graph shows the absolute number of cells from one out of three independent experiments. **D**. Percentage of AnnexinV+ PC-9 or A549 cells treated for 48 h with 1 μg/ml cisplatin in the presence of conditioned media collected from activated HFL1.

To assess the physiological relevance of satellite transcripts in functions of cancer-associated fibroblasts, we monitored the growth of two lung cancer cell lines PC-9 and A549 plated on top of human fibroblasts with or without knock-down of repetitive satellite elements. Fibroblasts were primed with conditioned media from corresponding cancer cells before plating and cells were grown in low serum conditions for 4 days. Knock-down of HS2/HS3 in fibroblasts was sufficient to slow down the proliferation of tumor cells (Fig. 6A; Fig. S9A). We also monitored the growth of tumor cells in low serum media supplemented with supernatant collected from HFL1 activated with conditioned media from tumor cells and also treated with bleomycin. To this end, we found that soluble factors from HFL1 cells stimulated the proliferation of both cancer cell lines (Fig. 6B; Fig. S9B, C). Knock-down of satellite RNAs was sufficient to attenuate tumor-promoting properties of factors secreted from CAFs. In fact, it was not possible to distinguish whether such an effect depended on a reduced number of satellite RNAs in the secretome of fibroblasts or due to systemic downregulation in the expression of multiple inflammatory genes. In addition, depletion of satellite transcripts in fibroblasts sensitized both PC-9 and A549 cells to treatment with cisplatin likely due to cumulative delayed tumor growth and slightly higher rate of cell death (Fig. 6C). Apoptosis rate was marginally increased when tumor cells were treated with cisplatin in conditioned media from primed si-HS2/HS3-transfected HFL1 cells (Fig. 6D; Fig. S9F), however without significant difference in number of apoptotic AnnexinV+ tumor cells in direct contact with fibroblasts (Fig. S9E). Collectively, these results show that pericentromeric satellite transcripts are important to control the production of secreted mediators from activated fibroblasts that promote cancer growth.

## Discussion

**Fig. 7:**
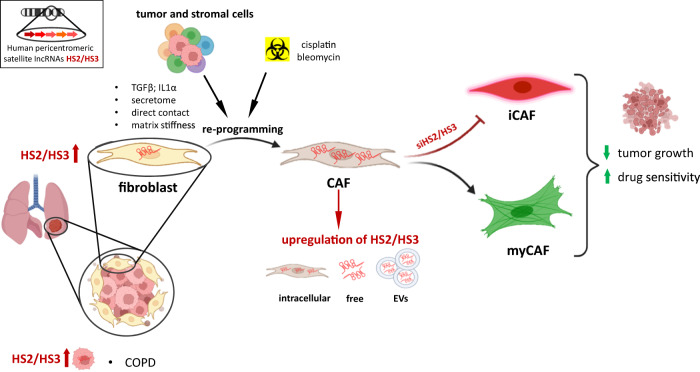
Graphical abstract. Transcription of pericentromeric satellite DNA is upregulated predominantly in cancer-associated fibroblasts in NSCLC or also in tumor cells in NSCLC patients with chronic obstructive pulmonary disease (COPD). Direct contact with tumor cells, matrix stiffness, TGFβ or IL1α in the tumor secretome re-program normal fibroblasts into CAFs and induce expression of satellite lncRNAs located in the nucleus, in the cytoplasm or secreted as free lncRNAs or packed in extracellular vesicles (EVs). HS2/HS3 is induced in both types of activated fibroblasts: myofibroblastic (myCAFs) and inflammatory (iCAFs). HS2/HS3 RNAs regulate the development of an inflammatory phenotype of CAFs supporting tumor growth. Depleting HS2/HS3 transcripts with siRNA impairs pro-tumorigenic functions of iCAFs, but not myCAFs, suppressing the proliferation of tumor cells and sensitizing cancer cells to chemotherapeutics.

In recent years, the transcription of pericentromeric DNA in cancer has been discovered [29, 30, 32]. Early studies demonstrated that the level of pericentromeric satDNA transcription in tumor tissue was higher than in normal tissue, such as in pancreatic and lung cancer of mouse and human origin [29]. However, at that time, single-cell NGS transcriptome sequencing had not been fully developed, which was a major obstacle for the detailed analysis of pericentromeric DNA transcription in tumor subpopulations. Most of the studies aimed to understand the role of satRNAs specifically in cancer cells [52]. Unfortunately, all these data do not address the impact of the tumor stroma. Cancer cells are embedded in a complex ecosystem of stromal cells. Fibroblasts, mesenchymal stromal cells, endothelium and other cells turn into CAFs in the TME [40]. The process of the activation of the CAF phenotype in mesenchymal stem cells and fibroblasts is accompanied by the acquisition of a senescence phenotype [53–55]. We showed that direct contact with tumor cells, TGFβ, IL1α or cytostatic drugs induced activation of CAFs that correlated with the expression of satDNA and the formation of a secretory senescence phenotype which was reduced by knock-down of satellite RNAs (graphical abstract, Fig. 7). Thus, conversion of stromal fibroblasts to CAFs with associated senescence and transcription of satellite lncRNA might be two tightly linked events leading to novel secretory features of CAFs. Although available studies are still limited, it is assumed that myCAFs exert tumor-restraining functions, whereas iCAFs rather exhibit tumor-promoting roles. Hence, targeting inflammatory CAF subtype might be more promising for therapeutic approaches than broadly and non-specifically depleting all kinds of cancer-associated fibroblasts. Our data shows that pericentromeric satellite RNAs express in both types of activated fibroblasts myCAFs and iCAFs in vitro and in situ; however, they regulate the development of an inflammatory phenotype of CAFs selectively. Depleting HS2/HS3 transcripts in fibroblasts did not significantly affect myCAF features that might possess antitumor functions (Fig. 7). Since HS2/HS3 tandemly repeated DNA represents about 3% of the genome, their transcripts can be highly present intra- and exracellularly or even in blood plasma. As a consequence, HS2/HS3 lncRNAs might have a tremendous impact on the physiology of cells and organs. Further development of methods to eliminate satellite transcripts during tumorigenesis might specifically reduce tumor inflammation mediated by cancer-associated fibroblasts.

SatRNA content in tumors might be different depending on several factors: tumor origin, tumor type, stage of tumor progression, metastasis; moreover, the level of transcription varies between individuals [29, 30]. In our study, the level of transcription in lung cancer cells was different in patients with or without COPD (Fig. S4). COPD is often associated with the smoking status of the patient, which in turn induces additional damage to the tissue. We demonstrated that transcription of satDNA in fibroblasts was induced by cisplatin and bleomycin (Fig. 2E, F) which could be a consequence of DNA damage [56, 57]. That might also explain an increased level of satellite transcripts in lung epithelial cells from cancer tissue with COPD versus no COPD. Ting et al. [29] observed a high level of pericentromeric DNA transcription in lung and pancreas tumors. However, the variation of TPM between samples was 1000-times or even more. Later Greenbaum and his team [30] demonstrated that a high level of HS2 transcription in colon and pancreatic cancers correlated with low levels of CD8 + lymphocytes and poor prognosis, while a low level of HS2 transcription was associated with a better prognosis in solid cancers. The authors suggest that in late-stage tumors, where abundant repetitive element expression is associated with failure of tumor suppressors, the large-scale transcription of many “non-self” repetitive elements has been co-opted by the tumor’s evolution to maintain an advantageous inflammatory state. The aberrant expression of satDNA either in tumor cells [31] or in CAFs (our study) may be one of the causes of inflammation in tumor tissues, subsequently leading to exhaustion of CD8 + T cells and a decrease in T-cell immune response demonstrated by Tanne et al. [31]. These observations led to the assumption that satRNA could be involved not only in the nuclear re-programming, but in other processes in the cytoplasm or in the extracellular space mediating cell-to-cell crosstalk. In the human lung adenocarcinoma samples used in our study, the hybridization signals showed cytoplasmic localization and were detected in CD63-positive vesicles. In line with our findings, EV produced by Ewing sarcoma were enriched with TR pericentromeric transcripts [58]. In the group of tumor patients, HS2/HS3 transcript levels were significantly elevated in plasma exosomes from patients with metastatic compared to those with localized disease or healthy individuals. Similarly, HS2 RNA sequences were detected at a higher level in sera of pancreatic ductal adenocarcinoma (PDAC) patients in soluble and exosomal fractions, suggesting that satellite transcripts could be used as screening markers for cancer patients [59]. Evdokimova et al. [58] provided evidence that despite their apparent lack of coding or replicative potential, HS2 transcripts are further propagated in recipient MRC5 fibroblasts derived from lung tissue and then released in their respective EVs that are capable of “infecting” freshly plated cells. Similar cell-to-cell transfer of satellite transcripts was demonstrated in our study. The HS2 transcripts are capable of inducing antiviral IFN-driven responses triggered by exosomes in the recipient cells, including myeloid progenitors and fibroblasts, due to pathogen-like motif patterns (i.e., high CpG content in AU-rich contexts as shown for an HS2 sequence). Indeed, high expression of repetitive elements, including LINEs, SINEs, and LTR/ERVs (long terminal repeats/endogenous retroviruses) correlated with an upregulated IFN pathway in PDAC with poor prognosis [60]. Although there is no published data yet, it is also possible that cytoplasmic satellite transcripts might trigger cGAS–STING pathway [61] as a major driver of inflammatory diseases. We also speculate that satDNA transcripts produced during carcinogenesis can exit a cell, like various other secreted small RNA [62], and act on other cells, generating a proinflammatory microenvironment which ultimately promotes tumor growth.

Thus, our study showed an increase in the level of pericentromeric RNA in a tumor due to activation of its transcription in TME cells, namely, cancer-associated fibroblasts (Fig. 7). Contact of mesenchymal stem cells (MSC) and fibroblasts with tumor cells can also lead to a change in the transcriptional activity of the non-coding part of the genome. These lncRNAs can serve as immunostimulatory “self-agonists” and directly activate myeloid immune cells to produce proinflammatory cytokines. That in turn can lead to suppression of an antitumor immune response as was suggested by Solovyov et al. [30] and Tanne et al. [31] for colorectal and pancreatic tumors. We claim that the transcription of pericentromeric DNA might be a general feature of cancer-associated fibroblasts noted in a broad range of human cancers. Extracellular satellite transcripts secreted by CAFs either in the form of free RNA or exosome-packed should be further studied for their functional role in tumor inflammation and immunity and extended to therapeutic interventions and diagnostic purposes using liquid biopsies.

## Material and methods

The detailed description of methods is given in Supplementary data.

### Oligonucleotide probes for FISH, DNA-RNA FISH, and immunoFISH

The following oligonucleotides were Cy3-labeled and used for FISH, RNA-FISH and immunoFISH: mouse MaSat probe 5′-AGGACCTGGAATATGGCGAGAAA-3′ [23, 36], human DYZ1 probe 5′-TCCATTCCATTCCATTCCATTCCATTCCATTCCATTCCATTCCATTCC-3′ [63]. The MaSat probe was designed using the following strategy. All the MaSat arrays were downloaded from whole-genome shotgun (WGS) databases. The frequency of each 12-mer subunit in MaSat repeat units was calculated [23]. The most frequent 12-mers were assembled into the MaSat oligonucleotide. The hybridization of probes to the pericentromeric regions of chromosomes was confirmed in mitotic spreads of mouse and human phytohemagglutinin-activated lymphocytes (Fig. S2).

### DNA-DNA, DNA-RNA FISH and immunoDNA-RNA FISH on cultured cells and histological sections

Human and mouse lung biopsies samples were fixed with 4 % formaldehyde (Sigma, Germany). Paraffine-embedded samples were cut into 5 µm sections. Before hybridization paraffin-embedded sections were deparaffinized in xylene and rehydrated and then dehydrated again in ethanol for FISH. Human and mouse cells grown in culture were fixed with 4% PFA. For DNA-FISH, cells or slices were denatured; for RNA-FISH and immunoRNA-FISH, the denaturation step was omitted. The Hybrizol VII (MP Biomedicals, USA) was mixed with an oligonucleotide probe and applied to cells or sections for overnight hybridization at 37 °C. Slides were then washed in 2xSSC at 41 ° C for 10 min, 1x SSC at RT for 10 min, and 0.5 x and 0.25x SSC for 5 min each. RNase treatment to prove the binding of the probes to RNA but not to DNA was carried out as described [63] after confocal imaging followed by dismounting of slides and washing them in PBS (3 × 10 min). If FISH was combined with immunostaining, the slices were hybridized, washed as described above, incubated in 5% BSA in PBS for 1 h and stained with a primary antibody (AB) overnight at +4 ^o^C (Table S6). The details of confocal imaging are described in Supplemental Materials and Methods Section.

### Activation of fibroblasts

For CAF phenotype induction, mouse lung fibroblasts and human fetal lung fibroblasts HFL1 were serum-starved for 2 d at 0.5% FCS-supplemented media and treated then with 10 ng/ml recombinant TGFβ1 (Dapcel Inc., USA), 10 ng/ml IL1α (Immunotools, Germany) for other 2 days or with 2 μg/ml cisplatin or 30 μg/ml bleomycin for 4 days. For stiffness experiment, HFL1 were drowned on 0.2 kPa or 50 kPa hydrogels coated with collagen I dishes (Petrisoft, USA). For coculture experiments, HFL1 cells were grown until fully confluent. PC-9 cells positive for CD326 were plated on top of HFL1 and grown for additional 4 days at 0.5 % FCS. PC-9 from cell mixture cells were depleted with anti-human magnetic CD326 beads (Miltenyi, Germany), a flow-through fraction containing fibroblasts was collected, tested to be negative for CD326 tumor cells and processed for RNA purification. For coculture experiments aimed to measure the proliferation rate of tumor cells, HFL1 was primed first with conditioned media from corresponding tumor cells for one day. The next day, tumor cells were labeled with cell tracker (Thermofisher, USA) and seeded in the density of 0.4 × 10^4^ per one well in 24 well plate on top of primed fibroblasts. Next day cells media was changed to 0.5% FCS or 5% FCS supplemented with 1 ug/ml cisplatin. 5 days later the number of labeled tumor cells was measured by flow cytometry (Cytoflex, BeckmanCulter, Germany). For growth of tumor cells in conditioned media (CM) from activated fibroblasts, lung cancer cells were seeded at density 1 × 10^4^ per one well in 24 well plate. Next day media was changed to 0.5% FCS with or without 50% of conditioned media collected from activated HFL1 cells; after 4 days cells were photographed and counted. To collect CM from activated HFL1, fibroblasts were primed with 50% of conditioned media from corresponding cancer cell cells (A549 or PC-9) in 0.5% FCS/DMEM, then treated (or left untreated) with 30 μg/ml bleomycin; one day later cells were intensively washed with PBS and grown in 0.5% FCS/DMEM for additional three days prior collection of CM.

### Computational analysis

Transcriptomes of various normal and tumor lung fibroblast cells were screened for the presence of DYZ1 sequence using publicly available single-cell RNA sequencing (scRNA-Seq) data [11]. A detailed description of the screening method is in Supplementary Materials and Methods. Briefly, reads belonging to each of seven fibroblast clusters along with the clusters of alveolar cells and cancer cells of the patients with adenocarcinoma and squamous carcinoma were extracted based on their barcode sequences. Each cluster was checked for the expression of corresponding marker genes (see below for fibroblasts; other markers are listed in Lambrechts et al. 2018 [11]). The authors revealed 7 clusters of fibroblasts. Cells from all clusters were overall only modestly enriched in tumors. However, cluster 1 was strongly enriched in tumors and cluster 6 was enriched in non-malignant samples [11]. Cluster 1 consisted of fibroblasts expressing *CTHRC1, VCAN, SULF1, COL10A1, POSTN, SFRP4, HTRA3, ASPN*. The fibroblasts expressing *COX4I2, HIGD1B, RGS5, GJA4, NDUFA4L2, PDGFRB, COL4A1, PTP4A3, COL4A2, LHFP, PPP1R14A, EGFL6, NOTCH3, PTN, COL18A1, ACTG2* were grouped into cluster 2. Cluster 3 was of poor quality without any detectable marker. The fourth cluster contained fibroblasts cells expressing PLA2G2A, SFRP2, and cluster 5 cells expressed *MMP3, SERPINE2*. The cells of cluster 7 were classified as the fibroblasts containing mRNA for *ALDH1A3, C3, CCL2, SOD2, TNFRSF12A, SLC20A1, NFKBIA, PTX3, DKK1, SERPINB2*. The normal lung fibroblasts were grouped into cluster 6. Their marker mRNA were those encoding *GPC3, MFAP4, A2M, CYR61, MACF1, GPX3, TIMP3, CFD, FIGF, LTBP4, SEPP1, ADH1B, CTGF, PTGDS, SCN7A, PRELP, MAMDC2, FHL1, DST, AOC3, INMT, NPNT, ELN, USP53, G0S2, FMO2*.

### Statistical analysis

GraphPad Prism software was used for statistical analysis. Graphs show mean values obtained with n technical or biological replicates, and error bars in all figures represent standard deviation (SD). *P-value ≤ 0.05; **P-value ≤ 0.01; ***P-value ≤ 0.001; ****P-value ≤ 0.0001; “NS” indicates nonsignificant with *P*-value > 0.05.

## Supplementary information

Supplementary text

Supplementary figures

## Data Availability

All data generated or analyzed during this study are included in this published article and its supplementary information files. Additional data related to this paper are available from the corresponding authors upon reasonable request.
