# Supplementary material for: Pericentromeric satellite lncRNAs are induced in cancer-associated fibroblasts and regulate their functions in lung tumorigenesis
Source: Cell Death Dis. 2023 Jan 12;14(1):19. doi: 10.1038/s41419-023-05553-1 (PMC9837065; doi:10.1038/s41419-023-05553-1)
Supplement: Supplementary file 1 — Supplementary text [file 41419_2023_5553_MOESM1_ESM.docx]

**Pericentromeric satellite lncRNAs are induced in cancer-associated fibroblasts and regulate their functions in lung tumorigenesis**

Natella I. Enukashvily^1,11*^, Nikita V. Ponomartsev^1,2,11^, Avanee Ketkar^3,4,5,11^, Roman Suezov^3,5^, Anna V. Chubar^1^, Andrey D. Prjibelski^6^, Daria D. Shafranskaya^6^, Sabrina Elmshäuser^4,5^, Corinna U. Keber^5,7^, Vera N. Stefanova^1^, Andrey L. Akopov^8^, Ursula Klingmüller^5,9^, Petra I. Pfefferle^5,10^, Thorsten Stiewe^4,5^, Matthias Lauth^3^, Anna I. Brichkina^3,4,5^*

^1^Institute of Cytology, Russian Academy of Sciences, 194064 St.-Petersburg, Russia.

^2^Institute of Molecular and Cell Biology, A*STAR, 138673 Singapore, Singapore.

^3^Philipps University of Marburg, Department of Gastroenterology, Center for Tumor- and Immune Biology, 35043 Marburg, Germany.

^4^Philipps University of Marburg, Institute of Molecular Oncology, 35043 Marburg, Germany. ^5^Member of the German Center for Lung Research (DZL), Philipps University of Marburg, Germany.

^6^Center for Algorithmic Biotechnology, St.-Petersburg State University, 199034 St.-Petersburg, Russia.

^7^Philipps University of Marburg, Institute of Pathology, 35043 Marburg, Germany.

^8^Pavlov First State Medical University, 197022 St.-Petersburg, Russia.

^9^German Cancer Research Center (DKFZ), 69120 Heidelberg, Germany.

^10^Philipps University of Marburg, Comprehensive Biobank Marburg CBBMR, 35043 Marburg, Germany.

^11^These authors contributed equally: Natella I. Enukashvily, Nikita V. Ponomartsev, Avanee Ketkar

* To whom correspondence should be addressed:

Anna Brichkina: Tel: +49(0)6421-28-25363; Email: anna.brichkina@staff.uni-marburg.de

Natella Enukashvily: Tel: +7(950)020-58-02; Email: n.enukashvily@incras.ru

## **Supplementary information**

**Results**

**Table S1.** **Transcripts per million (TPM) value for HS2/HS3 in human lung fibroblasts’ transcriptomes published and clusterized by Lambrechts et al. 2018^1^**

| **Fibroblasts cluster number in Lambrechts et al. 2018** | **Markers** | **Fibroblasts type** | **Normal tissue** | **Tumor periphery (middle part+edge)** | **Tumor core** |
| --- | --- | --- | --- | --- | --- |
| 6 | *GPC3, MFAP4, A2M, CYR61, MACF1, GPX3, TIMP3, CFD, FIGF, LTBP4, SEPP1, ADH1B, CTGF, PTGDS, SCN7A, PRELP, MAMDC2, FHL1, DST, AOC3, INMT, NPNT, ELN, USP53, G0S2, FMO2* | Normal lung fibroblasts ^1,2^ | 0.0000 | 0,0000+  48,5135 | 37.9269 |
| 1 | *CTHRC1, VCAN, SULF1, COL10A1, POSTN, SFRP4, HTRA3, ASPN* | CAF-Myofibroblasts ^2^ | 21.0300 | 11,7506+  17,1124 | 9.8714 |
| 2 | *COX4I2, HIGD1B, RGS5, GJA4, NDUFA4L2, PDGFRB, COL4A1, PTP4A3, COL4A2, LHFP, PPP1R14A, EGFL6, NOTCH3, PTN, COL18A1, ACTG2* | Pericytes ^2^ | 0.9747 | 8,6806+  2,3336 | 3.6371 |
| 4 | *PLA2G2A, SFRP2* | Stress-response CAFs. Pro-inflammatory normal fibroblasts ^1,2^ | 4.0800 | 5,5274+  3,9022 | 0.0000 |
| 5 | *MMP3, SERPINE2* | Activated catabolic CAFs ^2,3^), profibrotic ^4^ involved in idiopatic pulmonary fibrosis ^3^, associated with COPD ^5^. | 3.6400 | 4,3296+  2,5434 | 2.2069 |
| 7 | *ALDH1A3, C3, CCL2, SOD2, TNFRSF12A, SLC20A1, NFKBIA, PTX3, DKK1, SERPINB2* | Inflammatory ^1,2^ | 8.2100 | 12,0814+  0,0000 | 0.0000 |

**Table S2. Analysis of published**^1^ **human lung tumor transcriptomes: list of the most abundant HS2/HS3 transcripts that share homology with the DYZ1 probe.**

| **No** | **Transcript** |
| --- | --- |
| 1 | TAGAATGGAAAGGAATGGAATGAAATCAACCCGATTGGAATGGAAAGGAATGCAATGGAATGGAATAGAATGGAATGGACTGGAGTGGAATGGAATGAAATGAAATCAACCCGATTGGAATGGAATGGAATGCAATGTAATGGAATGGAATCAACTGTAAAGGAATGAAATGGAATGGAACGGAATGGAATGGAATGGAAAGGAATGGAATGGAATGGAATCAACCCGAGTGGAATGCAATGGAATGGAATGGAATGGAATGGAATCAACCCGAGTGGAACGGATTGCAATGGAGTGGAATGGAATGGAATGGCATGGAATGCAATGGAATCAACAAGAATGGACTGGAAGGGAATGGATTGGAATGGAATCGAACGGAATCAACCTGAGTAGAATGGAATGGAATGGAATGGAGTGAATGGAATGGAATGGAATGGAGTGAATGGAATGGAATGGAAT |
| 2 | TCCATTCCATTCCACTAGGGTTGATTCCATTCCATTCCATTCCATTCCATTCCGTTCCATTCCATTCCGTTCCATTTGATTCCTTTCCAGTTGATTCCATTCCATTCCATTGCATTCCATTCCATTCCAATCGGGTTGATTTCATTCCATTCCATTCCATTCTAGTCCATTCCACTCCATTCCATTCCATTCCATTCCATTCCTGTCCATTCCGTTCCATTCCATTCCATTC |
| 3 | TCCATTGCATTCCATACCTTTCGAGTCTATTCCGTTCTATTCCATTCTAGTCCATTCCATTCCATTAGAGTCCATTCCATTAAATTCCATTGTATTCCATTCGAGTCCATTCCATTCCATTTGGTTCCATTTGTGTCCATTCCATTCCATTTGAATCCATTCCATTCCATTTCTTTCGAGTCCATTCCATTCCATTCCATTCTATTCCATTCAAGTCCATTCCATTCCATTCAGTTCCATTCCATTAGAGTCCATCCCATTCAAGTCCATTCCATTCCATTCCATTCCATTCCATTCCAATGCATTCCATTGCATTCCTTTTGGATCCTTTCAATTCAACTGCATTCTAATTGAGTCCATTCCATTCCATTCATGTCCATTCCATTAGAGTCCATTCAATTCCATTCCATCCCATTC |

**Table S3***.* **Transcripts Per Million (TPM) value for HS2/HS3 in human lung myeloid cells’ transcriptomes published and clusterized by Lambrechts et al. 2018**^1^**.**

| **Myeloid cells cluster number in Lambrechts et al. 2018**^1^ | **Markers** | **Type of myeloid cells** | **Normal tissue** | **Tumor tissue** |
| --- | --- | --- | --- | --- |
| 1 | NA | macrophages | 4,0865 | 4,4503 |
| 2 | NA | macrophages | 6,5715 | 11,6370 |
| 3 | NA | macrophages | 0,0000 | 0,0000 |
| 4 | *ISG15, FAM26F, IFIT3, IFIT2, RSAD2, IFIT1, C15orf48, APOBEC3A, CCL8* | M1 macrophages (IFN pathway) | 6,24160 | 3,0994 |
| 5 | *CD1C, CD1E, PKIB, CD1A, FCER1A* | Langerhans cells | 18,0418 | 0,0000 |
| 6 | *FABP4, MCEMP1* | Alveolar macrophages | 28,5875 | 2,2610 |
| 7 | *S100A12, S100A9, S100A8, NAMPT, FCN1, IL1B, CD300E, EREG, CXCL8* | granulocytes | 0,0000 | 4,2476 |
| 8 | *CRIP1* | macrophages | 0,0000 | 0,0000 |
| 9 | *LST1, IFITM2, LYPD2, CFP, LINC01272, LILRA5* | Monocyte-derived dendritic cells | 0,0000 | 2,9367 |
| 10 | *F13A1, FOLR2, SLC40A1, HMOX1* | M2-like macrophages | 0,0000 | 0,0000 |
| 11 | *EEF1A1, ALDH2, HLA-DQA2, RBP4, C1QC* | Adipocytes-like macrophages, lipid metabolism | 0,0000 | 0,0000 |
| 12 | *CLEC9A, IDO1, LSP1, LGALS2, CPVL, CCR7, 1orf54, NAAA, S100B, CCL19, CCL17* | Cross-presenting dendritic cells | 28,8965 | 0,0000 |

**Table S4***.* **Transcripts Per Million (TPM) value for HS2/HS3 in human lung cells population transcriptomes published and clusterized by Lambrechts et al. 2018**^1^**.**

| **Cells populations in Lambrechts et al. 2018** | **Subpopulation (Markers if available)** | **TPM*** |
| --- | --- | --- |
| Fibroblasts | NLF (*GPC3, MFAP4, A2M, CYR61, MACF1, GPX3, TIMP3, CFD, FIGF, LTBP4, SEPP1, ADH1B, CTGF, PTGDS, SCN7A, PRELP, MAMDC2, FHL1, DST, AOC3, INMT, NPNT, ELN, USP53, G0S2, FMO2*) | 26,3644 |
|  | myCAF (*CTHRC1, VCAN, SULF1, COL10A1, POSTN, SFRP4, HTRA3, ASPN*) | 14,6174 |
|  | SR CAFs. Pro-infl. NF (*PLA2G2A, SFRP2*) | 3,4454 |
|  | AC CAF (*MMP3, SERPINE2*) | 3,0187 |
|  | Perycytes (*COX4I2, HIGD1B, RGS5, GJA4, NDUFA4L2, PDGFRB, COL4A1, PTP4A3, COL4A2, LHFP, PPP1R14A, EGFL6, NOTCH3, PTN, COL18A1, ACTG2*) | 2,9457 |
|  | Inflammatory CAF ( *ALDH1A3, C3, CCL2, SOD2, TNFRSF12A, SLC20A1, NFKBIA, PTX3, DKK1, SERPINB2*) | 5,5346 |
| Epithelial non-tumor cells | Cuboidal alveolar type 2 cells (*SFTPA1, SFTPA2, SFTPC, PGC*) | 2,5044 |
|  | Flat alveolar type 1 cells (*AGER, CAV1*) | 2,6519 |
|  | COPD-injured alveolar cells (*MMP7, CXCL14*) | 7,8116 |
|  | Respiratory epithelial cells (*EMP2, AGER, UPK3B, SPOCK2, CYP4B1, CAV1, RGCC, ICAM1*) | 1,3662 |
|  | Secretory club cells (*SCGB1A1, BPIFB1, TMEM45A, SCGB3A1, WFDC2, TSPAN8, CP, SAA2, MUC5B, LTF, PIGR, CXCL1, SCGB3A2*) | 2,8983 |
|  | Basal cells (*KRT17, KRT5, SYT8, MMP1, IGFBP6, FHL2*) | 5,2167 |
| Macrophages | macrophages | 4,3806 |
|  | macrophages | 4,6951 |
|  | macrophages | 9, 7311 |
|  | M1 macrophages (IFN pathway) (*ISG15, FAM26F, IFIT3, IFIT2, RSAD2, IFIT1, C15orf48, APOBEC3A, CCL8*) | 5,6774 |
|  | Langerhans cells (*CD1C, CD1E, PKIB, CD1A, FCER1A*) | 6,6726 |
|  | Alveolar macrophages (*FABP4, MCEMP1)* | 3,7009 |
|  | Granulocytes (*S100A12, S100A9, S100A8, NAMPT, FCN1, IL1B, CD300E, EREG, CXCL8*) | 9,8031 |
|  | Macrophages (*CRIP1*) | 2,6701 |
|  | Monocyte-derived dendritic cells (*LST1, IFITM2, LYPD2, CFP, LINC01272, LILRA5)* | 5,0386 |
|  | M2-like macrophages (*F13A1, FOLR2, SLC40A1, HMOX1*) | 5,1670 |
|  | Adipocytes-like macrophages, lipid metabolism (*EEF1A1, ALDH2, HLA-DQA2, RBP4, C1QC*) | 0,9063 |
|  | Cross-presenting dendritic cells (*CLEC9A, IDO1, LSP1, LGALS2, CPVL, CCR7, 1orf54, NAAA, S100B, CCL19, CCL17)* | 4,6951 |
| Cancer cells | Pat1_SC_COPD | 81,7671 |
|  | Pat2_SC_COPD | 75,3221 |
|  | Pat3_AD_noCOPD | 7,0660 |
|  | Pat4_AD_COPD | 32,4112 |

NLF – normal lung fibroblasts; myCAF – CAF with myofibroblast phenotype; SR CAF – stress-responsive CAF; AC CAF – activated catabolic CAF; COPD - Chronic obstructive pulmonary disease; Pat1_SC_COPD – Patient 1, squamous carcinoma, COPD; Pat2_SC_COPD Patient 1, scamous carcinoma, COPD; Pat3_AD_noCOPD - Patient 3, adenocarcinoma, no COPD; Pat4_AD_COPD - Patient 4, adenocarcinoma, COPD

* – Note that the sum of TPM values for a certain gene/transcript across different conditions is not equal to the TPM value when conditions are combined due to the design of TPM metric. The TPM sum of all genes in every particular experiment is equal to 1 million, which makes this metric non-additive, therefore the value in TPM column is not equal the sum of TPM values in columns ‘Normal tissue’ and ‘Tumor tissue’ from the Table S2.

**Supplementary Legends**

**Fig. S1***.* **Transcription of satellite RNA in mouse tumors.** **A** Representative image of H&E stained section of mouse lungs with tumors from Kras^G12D^ mice 10-14 weeks of age. **B** RT-qPCR analysis of MaSat transcription in lung tissues obtained from healthy mice (grey bars), lung tissue adjacent to cancer (black bars), and from lung tumors (red bars). Each sample is a pool of tissues from 3 individual mice (n=3). The fold change is plotted on the Y-axis. Data are mean±SD. ** p<0.01; *** - p<0.001; ns – non-significant. **C** qPCR analysis of MaSat expression using different primers for cDNA synthesis. The same RNA obtained from adjacent and tumor tissues (pool of ≥3 pieces) was used as a template to synthesize cDNA with oligoDT, random hexamer or specific to mouse MaSat primers. Y-axis - fold change (n=3). Data are mean ± SD. *** - p<0.001

**Fig. S2. Patterns of distribution and localization of mouse and human pericentromeric DNA and RNA.** Patterns of distribution of mouse (**panel I**) and human (**panels II, III**) pericentromeric DNA and RNA on mitotic lymphocyte spreads and in lung sections. **Panel I.** On mitotic spreads, both probes (red) hybridized to pericentromeres of most chromosomes (I-A; II-A). The pattern of nucleic acids hybridization was different – MaSat DNA (I-B) resided in chromocenters (I-B; insert) as expected according to the published data; MaSat RNA (I-C) was localized in the area between chromocenters (I-C; insert). **Panel II.** In human, two patterns of RNA hybridization foci distribution were revealed – 1) intranuclear (panel II-B); 2) outside the nucleus (II-C). The hybridization foci were not revealed in samples treated with RNAse before (I-D, II-D) or after (III-B) the FISH step. **Panel III.** The same section is shown as in Appendix Fig S6B (tumor). The slide (III-A) was dismounted after the immunoFISH procedure and confocal imaging, washed from the mounting medium, treated with RNase (III-B) and subjected again to confocal imaging; the layer of immunostaining with the AB against cytokeratines (shown in Supplementary Fig S3B) was removed in panel III for better visualization of the HS2/HS3 transcripts. Nuclei are counterstained with DAPI. For each panel a representative image of no less than 10 scanned areas is shown. Scale bars – 5 µm in I-A, II-A-II-D; 50 µm in all other panels.

**Fig. S3 Human pericentromeric HS2/HS3 is transcribed in tumor fibroblasts and mesenchymal stromal cells and show extracellular localization.**   **A** Tumor sections were stained with a fibroblast marker, anti-vimentin AB (green) and hybridized to HS2/HS3 probe. **B** Tumor sections probed with HS2/HS3 (red) and co-stained with a mix of CD44 and CD90 ABs (green) to reveal mesenchymal stromal cells. **C** Tumor sections probed with HS2/HS3 (red) and co-stained with anti-CD68 (green), marking a broad spectrum of macrophage subpopulations. The nuclei in all images are counterstained by DAPI (blue). Scale bars (50 µm) are shown in the images. Total magnification – x400. **D, E** Two patterns of HS2/HS3 extracellular localization (red) in human lung sections co-stained with cytokeratin (green): **D** transcripts are localized close to the cellular membrane and **E** in the extracellular matrix. The nuclei in all fluorescent images are counterstained by DAPI (blue). The correspondent phase contrast images are shown to the right. For each panel a representative image of no less than 10 scanned areas is shown. Scale bars (30 µm) are shown in the images. Total magnification – x400.

**Fig. S4. Transcription of HS2/HS3 in lung epithelial cells in squamous carcinoma (SC) or adenocarcinoma (AD) with or without COPD. A** Raw reads (ArrayExpress ,Acc No: E-MTAB-6149 and E-MTAB-6653) obtained by Lambrechts et al. 2018^1^ were used for transcriptomes reassembly and transcript~~s~~ quantification all cells from tumor lungs (Table S3) in squamous carcinoma (SC) or adenocarcinoma (AD) with or without chronic obstructive pulmonary disease (COPD). The fibroblast subpopulation labels are the same as in Fig. 2A of the main text. All other subpopulation labels are the same as in Table S3. Data of quantification is shown as TPM (transcripts per million) value plotted on the Y-axis. **B** Normal and tumor lung tissue sections were stained with the E-cadherin AB (**a**, green), the CKMNF116pan-cytokeratinAB (**b**, green), αSMA AB (**c**, green) and hybridized to HS2/HS3 probe (red). The corresponding phase-contrast images are shown. The nuclei in all images are counterstained by DAPI (blue). For each panel a representative image of no less than 10 scanned areas is shown. Scale bars (50 µm) are shown in the images. Bright yellow fibers in B are autofluorescence signals from extracellular matrix fibers. Total magnification – x400. **C** qRT-qPCR analysis of HS2/HS3 transcripts in primary fibroblasts established from resected tumors of two patients with matching healthy fibroblasts. Data are mean from three independent experiments ± SD (n=3). Ns – non significant.

**Fig. S5. Trancription of HS2/HS3 during EMT transition in lung cancer cells.** Lung cancer cells A549 and H1975 were treated with 5 ng/ml TGFβ1 for 6 d. Satellite transcripts and selected gene markers of EMT (*SLUG*, *ZEB1*) and TGFβ pathway were validated by qPCR. The fold change in relative expression as f change is presented at the graph (n=3). Data from one out of two independent experiments for each cell line is presented at the graph.

**Fig. S6. Transcription of satellite DNA in tumor-associated macrophages.** **A** Raw reads obtained by Lambrechts et al. 2018^1^ were used for transcriptomes reassembly and transcripts quantification in myeloid cells (Table S2; data with TMP as zero were not included in the graph). **B** The level of RNA transcripts for HS2/HS3 and positive markers of activation/differentiation in non-differentiated monocytes (day 1 post-plating) and fully differentiated macrophages (day 8), in macrophages treated with IL4, with conditioned media (CM) from human lung cancer cells or with IFNγ+LPS or cocultured with human lung cancer cells. Fold change difference in expression is shown on the Y-axis (n=3; three independent donors). **C** The level of RNA transcripts for MaSat and corresponding markers of activation in mouse bone marrow-derived macrophages treated either with IL4, IFNγ+LPS or with conditioned media (CM) from mouse lung cancer cells. Y-axis - fold change or relative expression (n=3). Data are mean ± SD. * - p<0.05; ** - p<0.01; *** - p<0.001

**Fig. S7**. **Transcription of HS2/HS3 and activation markers in human macrophages.** The level of RNA transcripts in PBMC derived human macrophages treated with conditioned media (CM) from A549 lung cancer cells or in direct coculture with A549 cells (the same donors as at the Fig. 3B). Genes were sub-grouped into M2-markers, M1-markers: inflammation, IFNγ-response or T-cell response. Fold change difference in expression is shown on the Y-axis (n=3). Data from one out of two independent experiments are presented at the graph. Data are mean ± SD. * - p<0.05; ** - p<0.01; *** - p<0.001.

**Fig. S8 Cytokine array of relative levels for cytokines/chemokines in conditioned media from bleomycin treated HFL1 cells.** A membrane-based antibody array of human cytokines (ARY022B, R&D Systems, USA) was used to determine relative levels of cytokines in conditioned media of si-scrambled and si-HS2/HS3 transfected fibroblasts and treated with bleomycin for 4 d. Photo of membranes used for quantification is presented. Number of cytokines differently present in the media is presented at the table (right).

**Fig. S9.** **HS2/HS3 satellite transcripts regulate pro-tumorigenic functions of CAFs. A** A549 and PC-9 cells were grown for 4 d in 0.5% FSC media in 2D contact coculture with HFL1 cells transfected either with si-scrambled (si-scr) or si-HS2/HS3 (si-HS3) RNAs. Absolute number of cells from one representative experiment is shown at the graph. **B** Representative photos or absolute number (**C**) of A549 or PC-9 tumor cells grown in the presence of conditioned media collected from activated HFL1 cells or pre-treated with bleomycin. **D.** A549 and PC-9 cells were grown for 4 d in 2D-coculture with HFL1 cells transfected either with si-scr or si-HS2/HS3 in the presence of 1 μg/ml cisplatin in media with 5% FCS. The graph shows percentage of AnnexinV+ tumor cells from one out of three independent experiments. **E**. Percentage of AnnexinV+ PC-9 or A549 cells pre-treated for 6 hrs with 1 μg/ml cisplatin in conditioned media from HFL1 cells, harvested and plated and grown with si-scr or siHS2/HS3-deficient HFL1 for 48 hrs.

### **Supplementary methods**

### **Animal models**

All animal experiments performed in this study received approvals from corresponding local authorities: Institute of Molecular and Cell Biology, Animal Safety and Use Committee (A*STAR, Singapore), German Animal Welfare Act (Deutsches Tierschutzgesetz). Transgenic mice with somatic activation of mutated *K-ras^G12D^* ^6^ and mice deficient for p53 were obtained from the Jackson laboratory (Bar Harbor, USA). Mice with lung cancer were euthanized at age of 10-14 wk. Mice with advanced tumors were euthanized once they developed critical conditions due to aggressive lung cancer (33-47 wk of age for wild type p53; p53-deficient - 11-19 wk old). Lungs were perfused through the trachea with PBS and excised. Visible tumors and the adjacent tissues were excised separately and used for RNA purification and subsequent qPCR. Each sample represents a pool of 3 or more individual tumors or adjacent tissue from one lung. In some cases (Supplementary Fig. 1A) each sample represents a pool of lesions from 3 or more mice. Bone marrow-derived macrophages and primary lung fibroblasts were obtained from C57BL/6J mice of 8-12 weeks old.

**Human material**

Approval for work with NSCLC biopsies was granted by the Ethics Committee of Philipps-University of Marburg (ethical votes 88/17 with the amendment and 05/19). Samples were processed and stored in Marburg Biobank CBBMR. The biopsy from resected lung tissue of the patients were obtained from untreated patients for diagnostic purposes. The samples for histology studies were fixed with 4% paraformaldehyde, embedded in paraffin, according to the standard protocols. Paraffin sections (5 µm) were sliced and put onto highly adhesive slides (Thermofisher, USA).

### **Cell culture and primary cells isolation**

Mouse fibroblasts were obtained from the lungs of C57BL/6 mice according to the protocol published in Brichkina et al. 2016^7^. Briefly, lungs were inflated with PBS and digested in DMEM supplemented with 2 mg/ml collagenase IV (Invitrogen, USA), 1 mg/ml dispase (Gibco, USA) and 0.1 mg/ml DNase I (Roche, Switzerland) at 37 ° C for 30 min with shaking at 100 rpm. Cells were then filtered through a 100 μm cell strainer, pre-treated with the hypotonic red cell lysis buffer (155 mM NH_4_Cl, 12 mM NaHCO_3_, and 0.1 mM EDTA) for 2 min and plated on 10 cm Petri dish in DMEM/F12 supplemented with penicillin-streptomycin and 15 % FCS (Sigma). If lungs are pooled from 3 mice, cells were plated on 15 cm Petri dish. Cells after the second passage were used for the experiments. Bone marrow-derived macrophages were obtained as follows. Femurs from the C57BL/6 mice were flushed with the RPMI medium. The cells were treated with the red blood cells lysis buffer for 2 min, plated on two 6 cm dishes (for one mice; if mice are pooled, number of dishes is increased proportionally) in the RPMI-1640 medium (ThermoFisherSceintific, USA) supplemented with 10 % FCS also containing penicillin, streptomycin and 50 ng/mL M-CSF (Immunotools, Germany) for four days to differentiate into macrophages. For activation, differentiated macrophages were treated for 24 hrs with 20 ng/ml IL4 (M2; Immunotools, Germany), 10 ng/ml IFNγ (Immunotools, Germany) + 100 ng/ml LPS (M1; Sigma, USA), or conditioned media (CM) collected from primary mouse lung cancer cells established from K-ras^G12D^/p53-/- mice, as previously reported by Brichkina et al. 2016^7^. CM was centrifuged prior to use for 5 min at 300 g to remove the cell debris, but preserve extracellular vesicles.

HFL1 line of human non-immortalized fetal fibroblasts was provided by Prof. Holger Sültmann (DKFZ, Germany). Cells were grown in F12 medium supplemented with 10 % of FCS (Aprotech, USA) and penicillin/streptomycin (Thermofisher Sci., USA). A549 and PC-9 cell lines derived from human lung adenocarcinoma (Sigma, Germany) were used for co-culturing with HFL1 cells. INC-049 early passage cell culture of human lung fibroblasts was obtained from the Cell Technologies Center Pokrovsky (St. Petersburg, Russia) and used for proliferation rate experiments using xCELLigence real-time cell analysis (RTCA) dual purpose (DP) system. Early passage (p. 1-4) cell cultures of human lung CAF and normal fibroblasts from patients with lung adenocarcinoma were obtained from Cell Technologies Center Pokrovsky (St. Petersburg, Russia).

**Isolation of human monocyte-derived macrophages (MDM)**

Peripheral blood mononuclear cells were obtained from the buffy coat of healthy adult volunteers of the Marburg University Clinic. Mononuclear cells were isolated by density gradient centrifugation in Lymphocyte Separation Medium 1077 (Capricorn Scientific, Germany), further plated on 10 cm dish and purified by adherent cell positive selection. Attached monocytes were cultured in RPMI-1640 supplemented with 1% Na-pyruvate, 1% Penicillin-Streptomycin solution and 5 % human AB serum (Sigma, Germany) for 7 days. Depending on the experimental goal, monocytes from 3-5 donors either were pooled together or cells from each donor were plated individually. For activation, MDMs were treated with 20 ng/ml IL4 (Immunotools, Germany), or with 10 ng/ml IFNγ + 100 ng/ml LPS, or with conditioned media collected from human lung cancer cells T27 (published in Brichkina et al., 2016^7^). For coculture, at day 8 T27 lung cancer cells were plated on top of macrophages initially in DMEM-F12medium supplemented with 2% FBS, 1% Penicillin-Streptomycin solution, B27 (Gibco, USA), 2 ng/ml EGF (Immunotools, Germany), which was then changed to DMEM/F12 medium with 0.5% FBS the next day. Cells were grown in coculture for 3 days after which the tumor cells were trypsinized for 2 mins and washed away with PBS. The underlying macrophages were then harvested for RNA purification and subsequent cDNA synthesis and RT-qPCR.

**RNA expression and quantitative real-time PCR**

Cells or excised lung tumors were harvested and processed for total RNA purification using RNAeasy kit (Qiagen, Germany or Macherey-Nagel, Germany) according to the manufacturer’s instructions, the samples were pre-treated with DNase (Qiagen, Germany). For lung tumors, each sample was a pool of ≥3 tumors from one mouse, or ≥3 tumors from different mice (variation will be described in the legend). cDNA was synthesized using SuperScriptVilo (Thermofisher, USA) or iScript (Bio-Rad, USA) kits with either oligo-dT or random primers or with a mix of these primers (Fig. S1C). Quantitative PCR reactions were performed using SYBR Green Master Mix (Thermofisher, USA) with specific primers listed at Supplemental Table 1. Results were calculated as relative mRNA expression (2ΔΔCt) using *Actin* for normalization. Data shown as the mean ± SD.

**siRNA transfection.**

Scrambled siRNA (5’-AAUUCUCCGAACGUGUCACGU-3’) and HS2/HS3 inactivating oligonucleotides (5’-AAUGGAAUCGAAUGGAAUC -3’ and 5’- GAUUCCAUUCGAUUCCAUU- 3’) were designed using consensus sequences for satellite transcripts described earlier^8,9^ and synthesized by Sigma (Germany). Each nucleotide of the sequences was 2-O-Methylated to increase duplexes stability and protect from nucleases ^10^. siRNA oligonucleotides were transfected with RNAimax transfection reagent (Thermofisher, USA) according to the manufacturer's instructions. 48 h after, transfected HFL cells were used for corresponding experiments.

**SA-βgal staining**

HFL1 cells were transfected and grown in low serum DMEM for 2 days, afterwards treated with corresponding factors for additional 4 d. Cells were washed with PBS and stained according to the manufacturer (Cell senescence kit, Cell Signalling, USA).

**Cytokine array**

Conditioned media has been collected from HFL1 cells treated with 30 µg/ml bleomycin for 4 d and processed to determine relative level of cytokines using Proteome Profiler Human XL Cytokine Array Kit (ARY022B, R&D Systems, USA) according to manufacturer instructions. Conditioned media was diluted 1:2 with a provided diluent before applying to the membranes. Membranes were developed using ECL western-blotting substrate (Thermofischer, USA) and ChemiDoc MP Imaging System (BioRad, USA) with 4 min exposure. Profiles of mean spot pixel density were created using image analysis software (Image Lab, BioRad, USA).

### **Computational analysis**

Raw reads from data published by ^1^ and available in ArrayExpress under accessions E-MTAB-6149 and E-MTAB-6653.were polyA-trimmed using Cutadap ^11^. For additional quality control, reads were mapped to the Human GRCh38 reference genome with STAR aligner ^12^ and checked for DNA contamination using Qualimap2 ^13^. Trimmed reads were further assembled using rnaSPAdes ^14^ and the assembled transcripts were screened for the presence of the DYZ1 sequence using the special mode of the BLAST aligner ^15^ designed for short sequences (-task blastn-short option). For further analysis, only transcripts containing highly confident matches were selected (an exact match with a half of the DYZ1 sequence). To check for the presence of poly-A tails (that are often removed by the assembly software) reads were mapped back to these transcripts using STAR ^12^. As a result, polyadenylated transcripts were detected, clustered using UCLUST and a representative consensus sequence was computed for each cluster.

By using published barcode sequences, reads corresponding to each cell type cluster were extracted with in-house scripts. Consensus transcripts were quantified using Kallisto 40 for each fibroblast cluster separately. In addition, we estimated the total expression for three known reference HS2/HS3 sequences—X60726.1, S90110.1, X82942.1 to check whether the quantity trends for clusters consensuses corresponded to trends for already-known sequences. To estimate the abundance of the assembled transcripts, we converted coverage values reported by rnaSPAdes to RPKM (Reads Per Kilobase Million) values.

### **Microscopy**

Image acquisition was performed using an Olympus FV3000 confocal microscope (Olympus, Japan). To detect DAPI, FITC, Cy3 and Alexa 647, the 405, 488, 561, and 646 nm diode lasers were used for excitation respectively. The cells were sectioned in z-axis with a 0.8 mkm interval. At each slide, images of at least five random visual fields were taken. Images were processed using Olympus FV3000 built-in software. The images were quantified using Fiji software. Before processing, images were calibrated – the ratio physical dimensions/number of pixels was calculated. Images of a single channel were processed with Subtract and Unsharp mask instruments of the software and were further converted to grayscale. Using the Threshold tool, a threshold value of gray was set and the image was converted to black-and-white – the values below threshold were converted to black, above it – to white pixels. The nuclei were manually selected using Freehand selection tool. The number of white pixels per nucleus was calculated. A similar approach was used in Corel PHOTOPAINT software – the cell areas were manually selected and the number of pixels above a threshold in the channel of interest was obtained from the image histogram. In this case, the step of conversion to black-and-white was omitted (to eliminate possible signal loss during conversion). The results of both approaches were compared using one way ANOVA test. When no significant difference was observed, the Fiji results were used for chart plotting and statistical calculations.

**Annexin V staining.** A549 or PC-9 cells were plated on 24 well plate at density 1x10^4^ cells per well, next day cells were treated with 4 uM cisplatin in 5% FCS/DMEM media containing 50% of conditioned media collected from HFL1 cells and analyzed 48 hrs later with Annexin V kit (Biolegend, USA) according to the manufacturer. For culturing with HFL1 cells, tumor cells were treated with 4 uM cisplatin for 6 hours, harvested, labelled with cell tracker (Invitrogen, USA), plated on top of HFL cells growing in 5%FCS/DMEM and analysed 24 hrs later with Annexin V kit.

**Table S5. Primers for RT-qPCR**

|  | F- primer | R- primer |
| --- | --- | --- |
| MaSat | GACGACTTGAAAAATGACGAAATC | CATATTCCAGGTCCTTCAGTGTGC |
| *m-αSMA* | CCACCGCAAATGCTTCTAAGT | GGCAGGAATGATTTGGAAAGG |
| *m-Actin* | GCTGTATTCCCCTCCATCGTG | CACGGTTGGCCTTAGGGTTCAG |
| *m-Gapdh* | GAAGGTGAAGGTCGGAGTC | GAAGATGGTGATGGGATTTC |
| *m-Arg1* | GGCTTATGGTTACCCTCCCG | CAAGACAGGGCTCCTTTCAG |
| *m-IL6* | CCGGAGAGGAGACTTCACAG | GGAAATTGGGGTAGGAAGGA |
| *m-CXCL9* | TGTGGAGTTCGAGGAACCCT | AGTCCGGATCTAGGCAGGTT |
| *h-Chi3L1* | AAGAACAGGAACCCCAACCTG | TGCTGTTTGTCTCTCCGTCC |
| *h-CCL18* | TGCTCCTGTGCACAAGTTGG | CTGGGGGCTGGTTTCAGAAT |
| *h-CXCL10* | TGGCATTCAAGGAGTACCTCTC | TGATGGCCTTCGATTCTGGA |
| *h-COL11A1* | GACTATCCCCTCTTCAGAACTGTTAAC | CTTCTATCAAGTGGTTTCGTGGTTT |
| *h-ACTIN* | GGCCAGGTCATCACCATTG | GGATGTCCACGTCACACTTCA |
| HS2/HS3 | AGTCCATTCAATGATTCCATTCCAGT | AATCATCATCCAACGGAAGCTAATG |
| *h-IL6* | TTCCATCCAGTTGCCTTCT TG | TTGGGAGTGGTATCCTCTGTGA |
| *h-IL1β* | GCAGAAGTACCTGAGCTCGC | CTGGAAGGAGCACTTCATCTG |
| *h-COL4A1* | AGGATCTGTTGGTGGAATGGGCTTG | CCTGCCTGCCCTTTCTCTCCTTTTG |
| *h-COL1A1* | \| TCAGCAAGAACCCCAAGGACAAGAGG \| \| --- \| \|  \| | \| AGGAAGGTCAGCTGGATGGCCACAT \| \| --- \| |
| *h-FN1* | CAGTGGAATGCACCACAGCCATCTC | TGGTAGCTTCCTTCCAACGGCCTACA |
| *h-CCL20* | GCTGCTTTGATGTCAGTGCT | GCAGTCAAAGTTGCTTGCTTC |
| *h-THBS1* | CAATGCCACAGTTCCTGATG | CACAGCTCGTAGAACAGGAGG |
| *h-HAS2* | GTGGATTATGTACAGGTTTGTGA | TCCAACCATGGGATCTTCTT A |
| *h-ZEB1* | GTCAGCCCTGCAGTCCAAGAACCAC | CCGCATTTTCTTTTTGGGCGGTGTA |
| *h-SLUG* | CCAGACCCTGGTTGCTTCAAGGACA | TGCTCTGTTGCAGTGAGGGCAAGAA |
| *h-CD80* | GGGGAAATGTCGCCTCTCTG | GTGGATTTAGTTTCACAGCTTGC |
| *h-CD206* | CACGATCCGACCCTTCCTTG | GTCTCCGCTTCATGCCATTG |
| *h-CCL18* | TGCTCCTGTGCACAAGTTGG | CTGGGGGCTGGTTTCAGAAT |
| *h-MMP12* | CTGCTGTTCACGAGATTGGC | GGGTCTCCATACAGGGACTGA |
| *h-TNFα* | GTTGTAGCAAACCCTCAAGCTG | GAGGTACAGGCCCTCTGATG |
| *h-IDO1* | GATGTCCGTAAGGTCTTGCC | GCAGCTGCTATTTCCACCAA |
| *h-COX2* | GGAATGTTCCACCCGCAGTA | CATCTGGCCGAGGCTTTTCT |
| *h-IRF1* | CCA GAG AAA AGA AAG AAA GTC GAA GTC C | CAT GGC GAC AGT GCT GGA GTC |
| *h-GBP6* | GAA TCA TGG CTT CCC TCT GGG C | GAT CCA GGA GTC ATT CTT AGG GTC AC |
| *h-CXCL10* | TGGCATTCAAGGAGTACCTCTC | TGATGGCCTTCGATTCTGGA |
| *h-aSMA* | GACGAAGCACAGAGCAAAAGAG | TGGTGATGATGCCATGTTCTATCG |
| *h-IL8* | ACCCCAAGGAAAACTGGGTGCAGAG | CTGGCAACCCTACAACAGACCCACA |
| *h-CXCL1* | GCCTCAATCCTGCATCCCCCATAGT | GCCTCCTTCAGGAACAGCCACCAGT |
| *h-TMEPAI* | GTGCAACTGCAAACGCTCTT | CCACAGGCATCCTTCTGAGG |

**Table S6. The antibodies (AB) used for immunochemical staining**

|  |  |  |  |
| --- | --- | --- | --- |
| **Primary ABs** | **Antigen** | **Labelled cells** | **Application** |
| Anti mouse/human Vimentin (Novus; #NB300-223) | Mouse Vimentin | Mouse fibroblasts | Immunohistochemistry |
| Anti-Iba1 (WAKO; #019-19741) | Mouse and human, microglia\macrophage marker | M1, M2 and TAM | Immunohistochemistry, immunoDNA-RNA-FISH |
| Anti E-cadherin (Cell Signalling; #3195) | Mouse and human E-cadherin | Normal and tumor mouse epithelia | ImmunoDNA-RNA-FISH |
| Anti α-smooth muscle actin (aSMA)-FITC (Sigma #F3777) | Human and mouseα-smooth muscle actin | Human and mouse CAF, normal myofibroblasts | immunoDNA-RNA FISH |
| - Anti pancytokeratin~~e~~s CKMNF116 (Dako ; # MNF116) | Human cytokeratines | Human tumor epithelium | ImmunoDNA-RNA-FISH |
| Anti human CD63-Alexa 647 (Molecular probes; #231975) | Human CD63 antigen | Extracellular vesicles | ImmunoDNA-RNA-FISH |
| Anti-PDGFRB (Cell Signalling; #3169) | Human PDGFRB | Human and mouse lung fibroblasts | ImmunoDNA-RNA-FISH |

**Table S7. SiRNA sequences**

| **Gene** | **Sequence** |
| --- | --- |
| Si-scrambled  2'O-Methyl RNA | mAmAmUmUmCmUmCmCmGmAmAmCmGmUmGmUmCmAmCmGmU |
| si-HS2/HS3  2'O-Methyl RNA | \| mAmAmUmGmGmAmAmUmCmGmAmAmUmGmGmAmAmUmC \| \| --- \| \| mGmAmUmUmCmCmAmUmUmCmGmAmUmUmCmCmAmUmU \| |

**Supplementary references**

1 Lambrechts D, Wauters E, Boeckx B, Aibar S, Nittner D, Burton O *et al.* Phenotype molding of stromal cells in the lung tumor microenvironment. *Nat Med* 2018; **24**: 1277–1289.

2 Hanley C, Weise C, Parker R, Lopez MA, Taylor J, Kimbley L *et al.* Spatially discrete signalling niches regulate fibroblast heterogeneity in human lung cancer. *bioRxiv* 2020; : 134270-undefined.

3 Peyser R, MacDonnell S, Gao Y, Cheng L, Kim Y, Kaplan T *et al.* Defining the activated fibroblast population in lung fibrosis using single-cell sequencing. *Am J Respir Cell Mol Biol* 2019; **61**: 74–85.

4 Redente EF, Chakraborty S, Sajuthi S, Black BP, Edelman BL, Seibold MA *et al.* Loss of Fas signaling in fibroblasts impairs homeostatic fibrosis resolution and promotes persistent pulmonary fibrosis. *JCI Insight* 2021. doi:10.1172/jci.insight.141618.

5 Korytina GF, Akhmadishina LZ, Aznabaeva YG, Kochetova O V., Zagidullin NS, Kzhyshkowska JG *et al.* Associations of the NRF2/KEAP1 pathway and antioxidant defense gene polymorphisms with chronic obstructive pulmonary disease. *Gene* 2019. doi:10.1016/j.gene.2018.12.061.

6 Johnson L, Mercer K, Greenbaum D, Bronson RT, Crowley D, Tuveson DA *et al.* Somatic activation of the K-ras oncogene causes early onset lung cancer in mice. *Nature* 2001; **410**: 1111–1116.

7 Brichkina A, Bertero T, Loh HM, Nguyen NTM, Emelyanov A, Rigade S *et al.* p38MAPK builds a hyaluronan cancer niche to drive lung tumorigenesis. *Genes Dev* 2016; **30**: 2623–2636.

8 Valgardsdottir R, Chiodi I, Giordano M, Cobianchi F, Riva S, Biamonti G. Structural and functional characterization of noncoding repetitive RNAs transcribed in stressed human cells. *Mol Biol Cell* 2005; **16**: 2597–604.

9 Dobrynin MA, Korchagina NM, Prjibelski AD, Shafranskaya D, Ostromyshenskii DI, Shunkina K *et al.* Human pericentromeric tandemly repeated DNA is transcribed at the end of oocyte maturation and is associated with membraneless mitochondria-associated structures. *Sci Rep* 2020; **10**. doi:10.1038/s41598-020-76628-8.

10 Lennox KA, Behlke MA. Chemical modification and design of anti-miRNA oligonucleotides. Gene Ther. 2011. doi:10.1038/gt.2011.100.

11 Martin M. Cutadapt removes adapter sequences from high-throughput sequencing reads. *EMBnet.journal* 2011; **17**: 10.

12 Dobin A, Davis CA, Schlesinger F, Drenkow J, Zaleski C, Jha S *et al.* STAR: ultrafast universal RNA-seq aligner. *Bioinformatics* 2013; **29**: 15–21.

13 Okonechnikov K, Conesa A, García-Alcalde F. Qualimap 2: advanced multi-sample quality control for high-throughput sequencing data. *Bioinformatics* 2016; **32**: 292–4.

14 Bushmanova E, Antipov D, Lapidus A, Prjibelski AD. rnaSPAdes: a de novo transcriptome assembler and its application to RNA-Seq data. *Gigascience* 2019; **8**. doi:10.1093/gigascience/giz100.

15 Camacho C, Coulouris G, Avagyan V, Ma N, Papadopoulos J, Bealer K *et al.* BLAST+: architecture and applications. *BMC Bioinformatics* 2009; **10**: 421.
