# Supplementary material for: Pericentromeric satellite lncRNAs are induced in cancer-associated fibroblasts and regulate their functions in lung tumorigenesis
Source: Cell Death Dis. 2023 Jan 12;14(1):19. doi: 10.1038/s41419-023-05553-1 (PMC9837065; doi:10.1038/s41419-023-05553-1)
Supplement: Supplementary file 2 — Supplementary figures [file 41419_2023_5553_MOESM2_ESM.pdf]

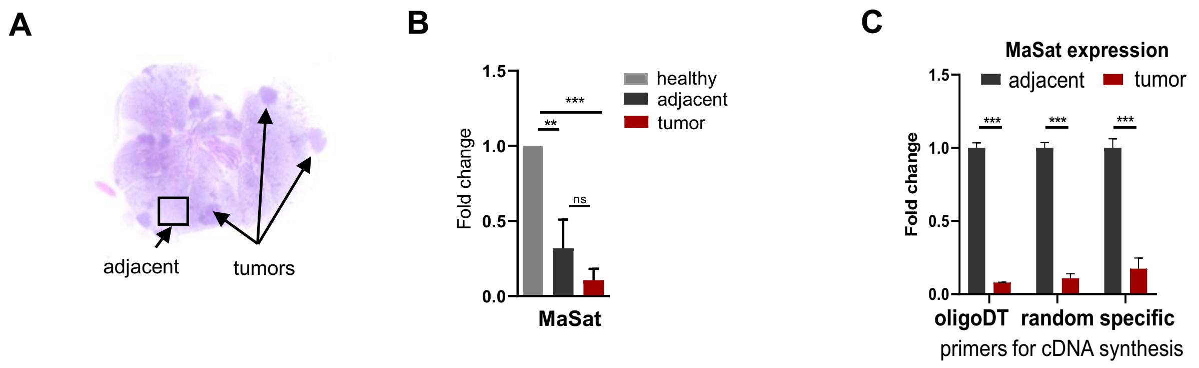
A
adjacent
tumors
B
1.5
1.0
0.5
0.0
Fold change
healthy
adjacent
tumor
***
**
ns
MaSat
C
MaSat expression
adjacent
tumor
1.5
1.0
0.5
0.0
Fold change
***
***
***
oligoDT
random
specific
primers for cDNA synthesis

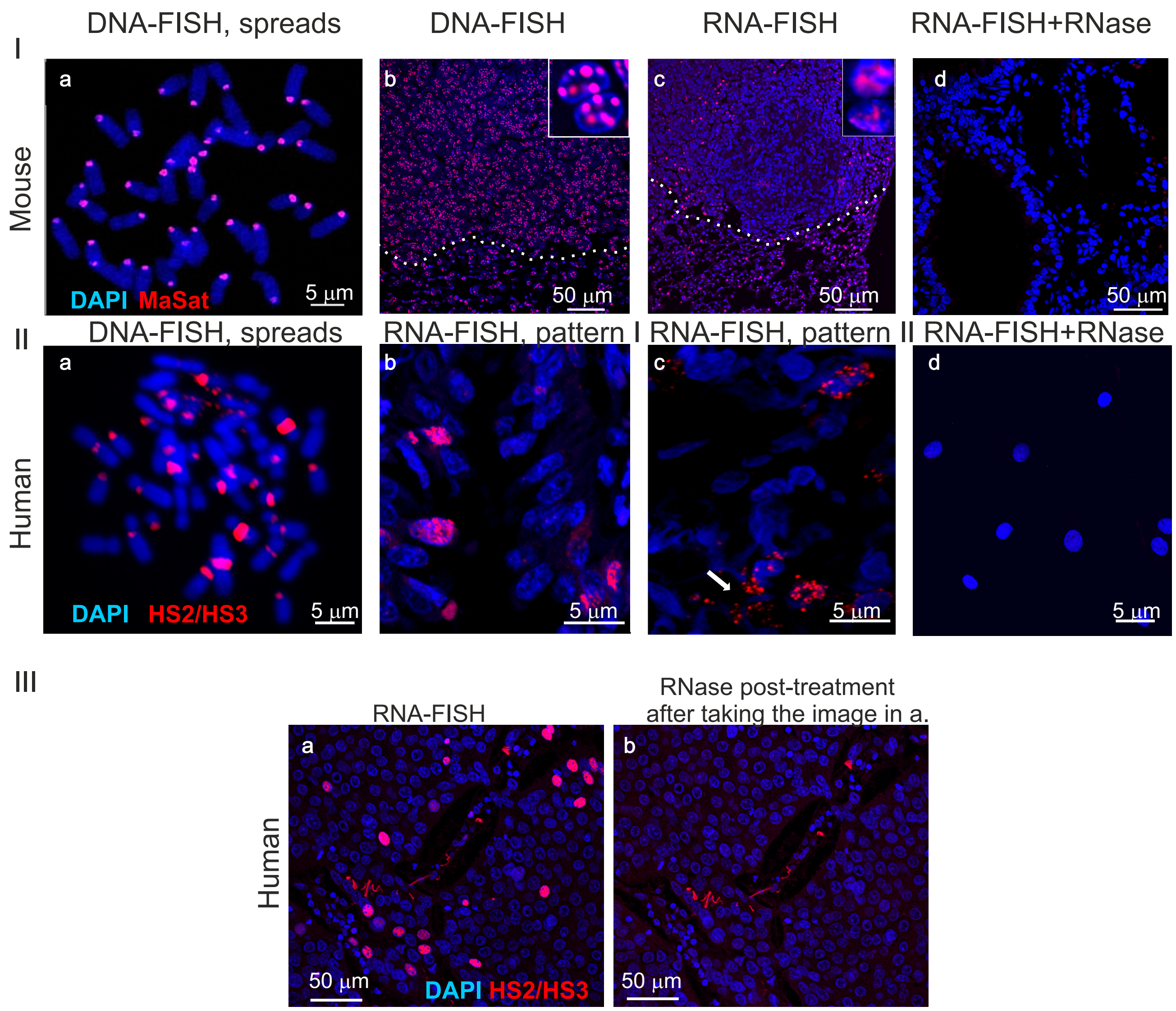
I
DNA-FISH, spreads
DNA-FISH
RNA-FISH
RNA-FISH+RNase
Mouse
a
b
c
d
DAPI MaSat
5 μm
50 μm
50 μm
50 μm
II
DNA-FISH, spreads
RNA-FISH, pattern I
RNA-FISH, pattern II
RNA-FISH+RNase
Human
a
b
c
d
DAPI HS2/HS3
5 μm
5 μm
5 μm
5 μm
III
RNA-FISH
RNase post-treatment
after taking the image in a.
Human
a
b
50 μm
DAPI HS2/HS3
50 μm

Enukashvily_Supplemental Fig. 3

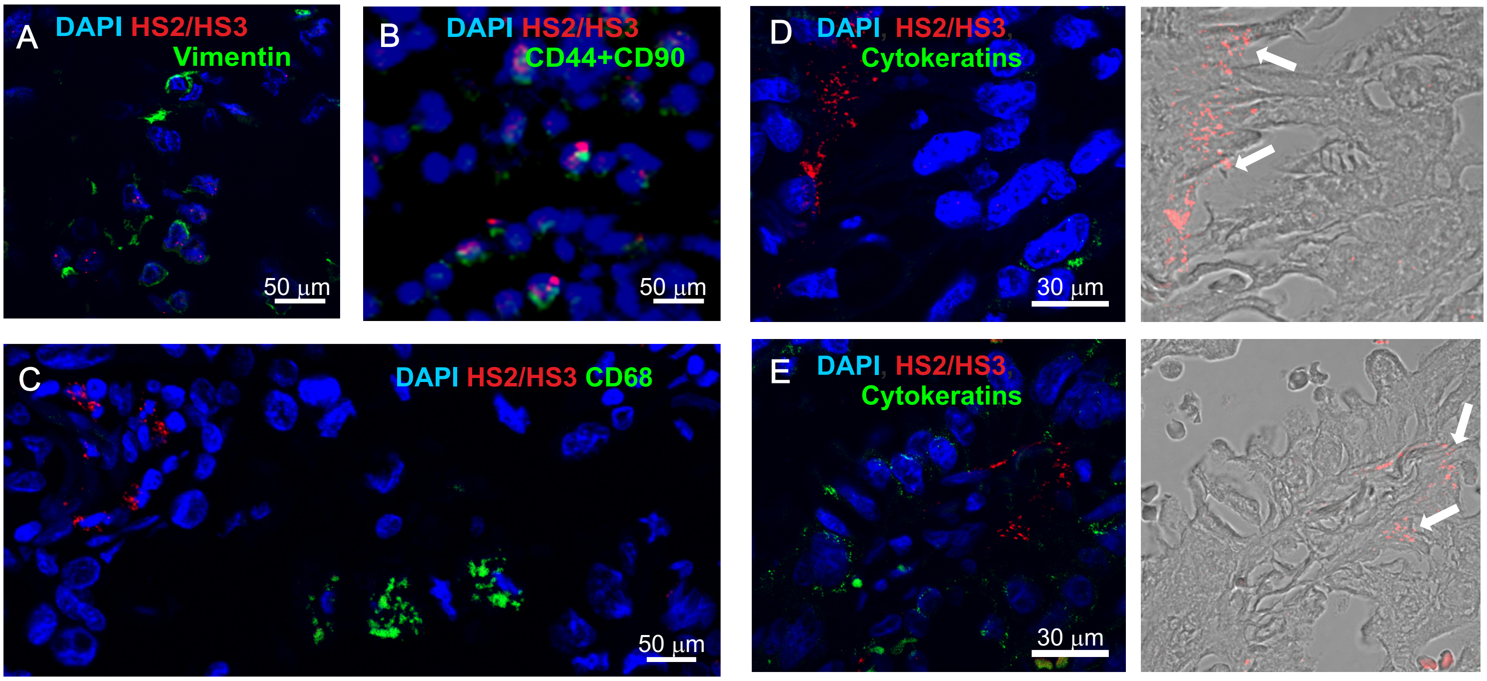

A

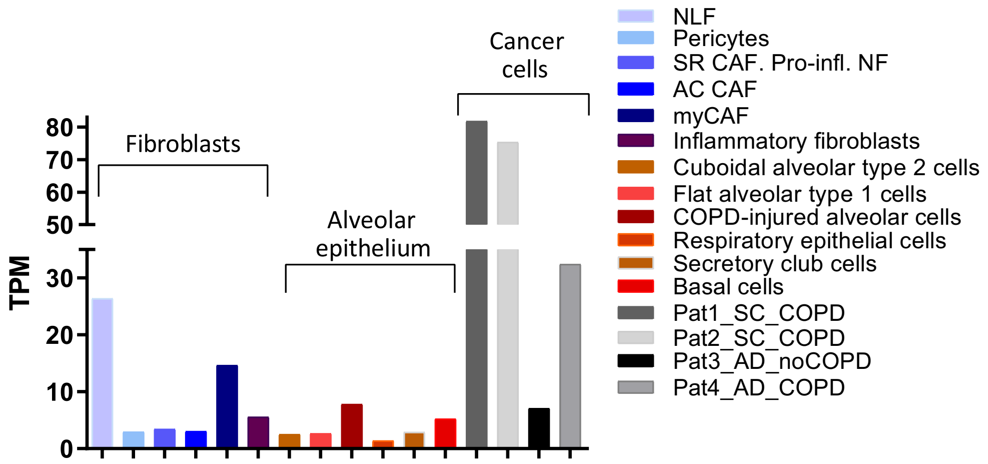

TPM
80
70
60
50
30
20
10
0
Fibroblasts
Alveolar epithelium
Cancer cells
NLF
Pericytes
SR CAF. Pro-infl. NF
AC CAF
myCAF
Inflammatory fibroblasts
Cuboidal alveolar type 2 cells
Flat alveolar type 1 cells
COPD-injured alveolar cells
Respiratory epithelial cells
Secretory club cells
Basal cells
Pat1_SC_COPD
Pat2_SC_COPD
Pat3_AD_noCOPD
Pat4_AD_COPD

B

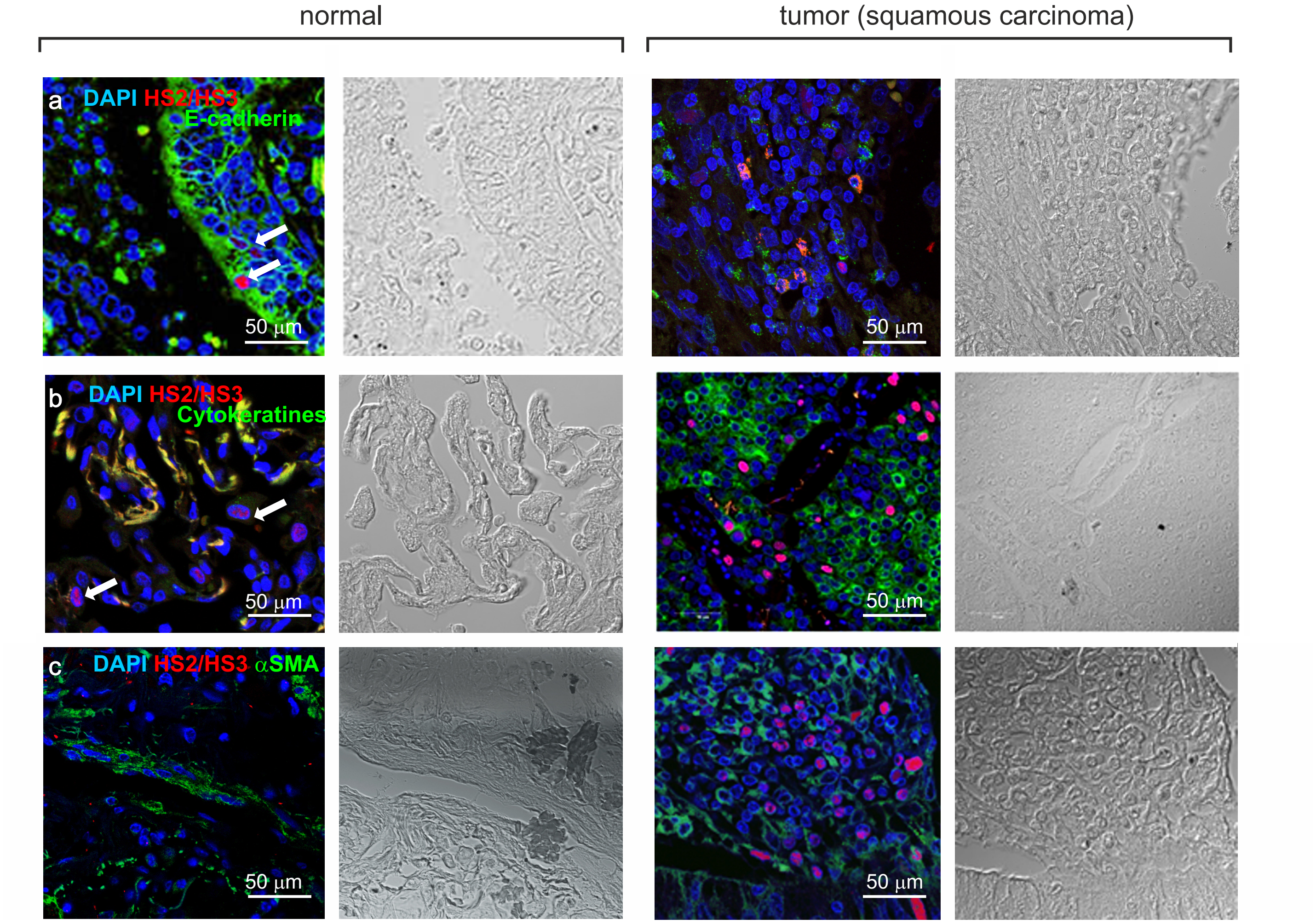

normal
tumor (squamous carcinoma)
a DAPI HS2/HS3 E-cadherin
b DAPI HS2/HS3 Cytokeratines
c DAPI HS2/HS3 αSMA
50 μm

C

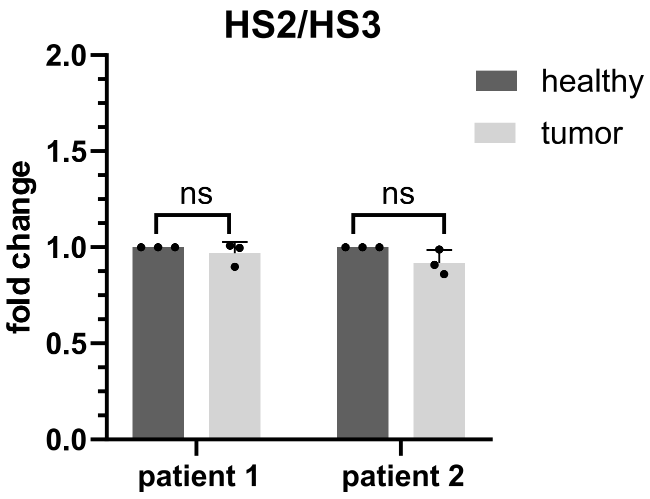

HS2/HS3
fold change
2.0
1.5
1.0
0.5
0.0
ns
ns
healthy
tumor
patient 1
patient 2

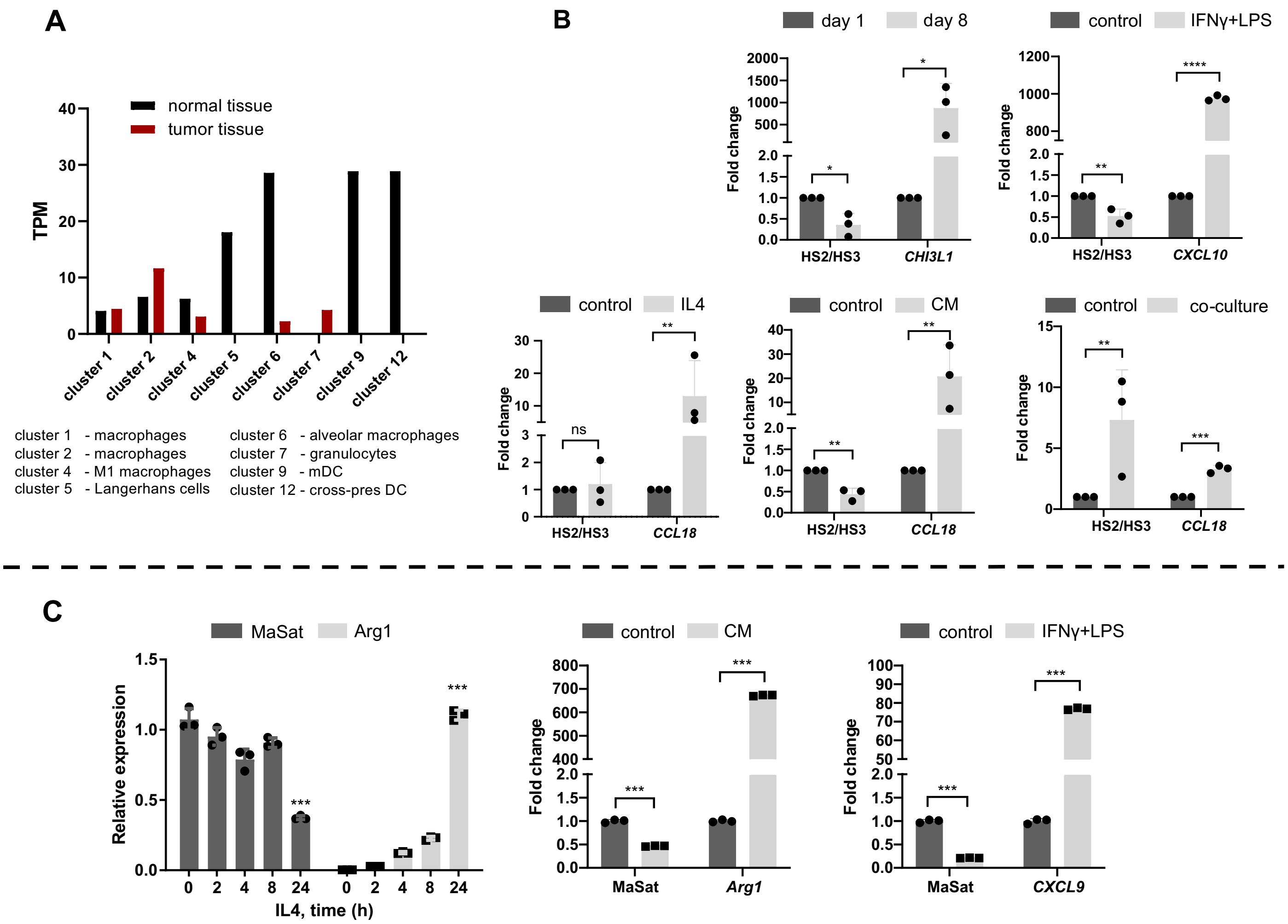
A
normal tissue
tumor tissue
TPM
cluster 1
cluster 2
cluster 4
cluster 5
cluster 6
cluster 7
cluster 9
cluster 12
cluster 1 - macrophages
cluster 2 - macrophages
cluster 4 - M1 macrophages
cluster 5 - Langerhans cells
cluster 6 - alveolar macrophages
cluster 7 - granulocytes
cluster 9 - mDC
cluster 12 - cross-pres DC
B
day 1
day 8
control
IFNγ+LPS
Fold change
HS2/HS3
CHI3L1
CXCL10
control
IL4
control
CM
control
co-culture
CCL18
C
MaSat
Arg1
Relative expression
IL4, time (h)
control
CM
control
IFNγ+LPS
MaSat
Arg1
CXCL9

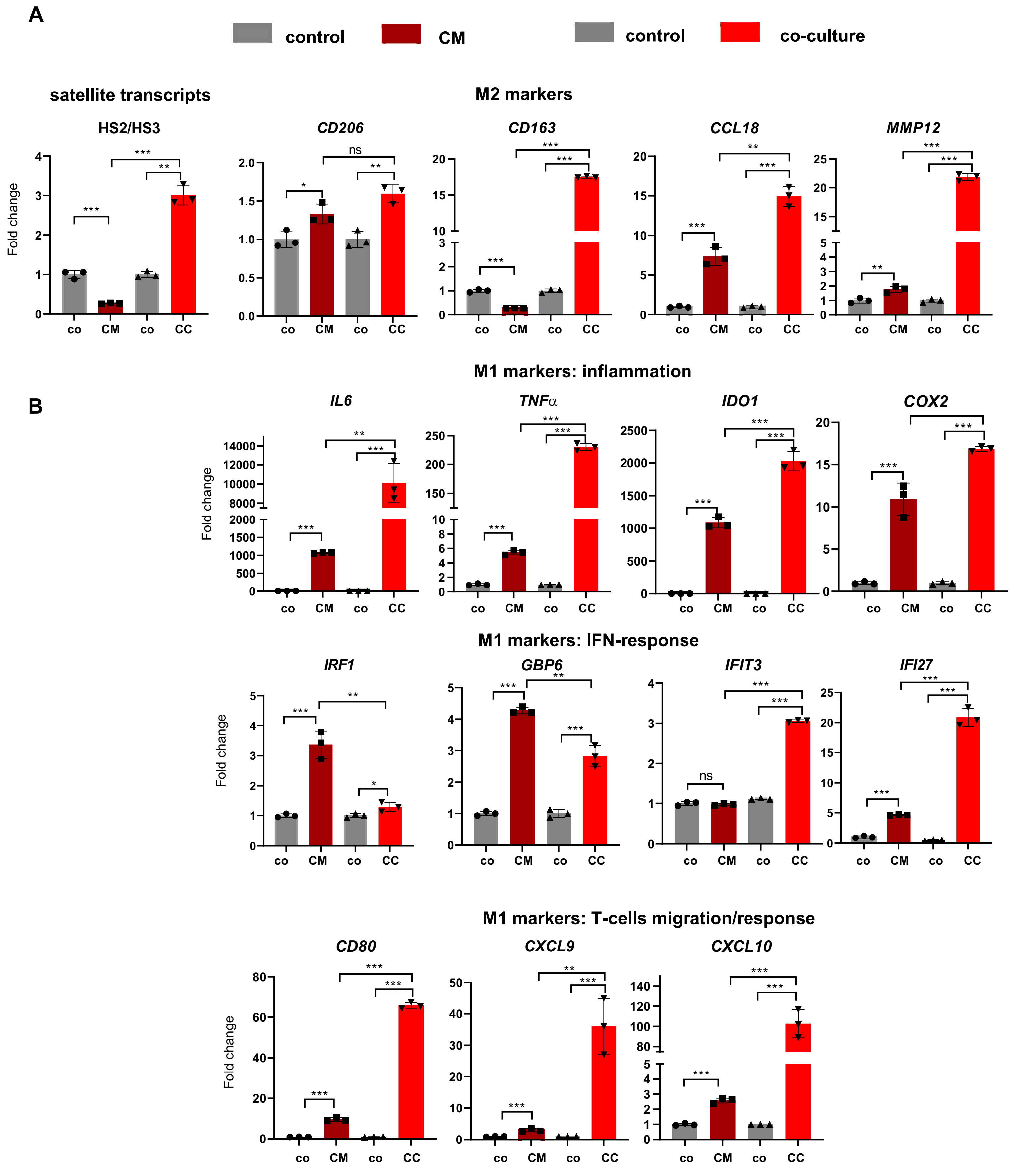
A
control
CM
control
co-culture
satellite transcripts
M2 markers
HS2/HS3
CD206
CD163
CCL18
MMP12
Fold change
co
CM
co
CC
B
M1 markers: inflammation
IL6
TNFα
IDO1
COX2
M1 markers: IFN-response
IRF1
GBP6
IFIT3
IFI27
M1 markers: T-cells migration/response
CD80
CXCL9
CXCL10

A

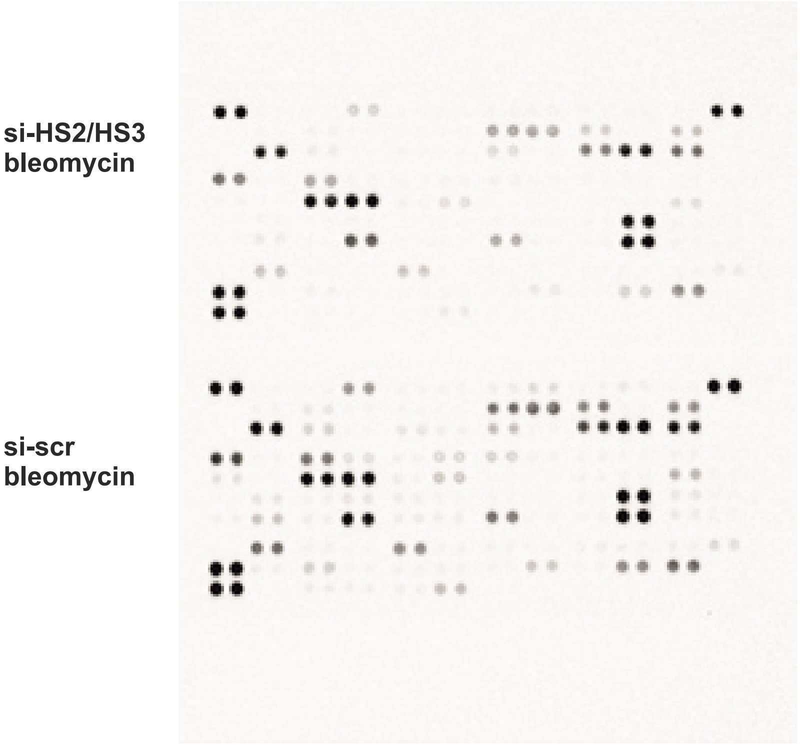

Cytokine array summary

**Total**	106
**up**	0
**non-changed**	69
**non-detectable**	9
**down**	28
